# Psychological distress, stigma, and intersectional marginalization in lesbian and bisexual cisgender women in Chinese higher education: a thematic analysis guided by minority stress theory

**DOI:** 10.3389/fpubh.2026.1893495

**Published:** 2026-08-28

**Authors:** Yan Liu

**Affiliations:** School of Humanities and Education, Xi'an Eurasia University, Xi’an, China

**Keywords:** Chinese higher education, gender inequality, internalized homophobia, intersectionality, lesbian and bisexual women, minority stress, psychological distress, reflexive thematic analysis

## Abstract

**Background:**

Lesbian and bisexual cisgender women in Chinese higher education institutions (HEIs) face compounded psychological distress at the intersection of gender- and sexuality-based stigma. Despite growing evidence of mental health disparities among LGBTQ populations in China, the mechanisms through which structural marginalization is internalized in educational settings remain poorly understood.

**Objective:**

To identify how structural stigma is translated into internalized psychological harm among lesbian and bisexual cisgender women in Chinese HEIs, through specific, theoretically specified mechanisms.

**Methods:**

Using a critical realist, reflexive thematic analysis design, I conducted semi-structured interviews with eight self-identified lesbian, bisexual, or same-sex attracted cisgender women at universities in four province-level regions of China. Analysis followed Braun and Clarke's (2006) six-phase approach, guided by Meyer's (2003) minority stress model, Hatzenbuehler's (2009) psychological mediation framework, and Crenshaw's (1989) intersectionality theory. Researcher positionality was integrated analytically throughout.

**Results:**

Five themes were identified: (1) awareness of structural inequality as anticipatory anxiety and cognitive vigilance; (2) institutional resource deprivation undermining academic self-efficacy; (3) enacted and internalized stigma and their psychological consequences, including shame, identity concealment, and self-reported depressive symptoms; (4) intersectional invisibility compounding distress; and (5) attenuated outcomes where community and institutional support are present. Three mediation pathways were consistently operative: social-cognitive, emotional regulatory, and interpersonal (Hatzenbuehler, 2009). No diagnostic instrument was administered; all psychological states reported are self-described and interpreted from interview accounts.

**Discussion:**

Distal stigma, internalized homophobia, culture-specific marriage pressure, and regional pathologization interact in complex ways to shape distress, disrupted identity development, and academic disengagement. I examine the limits of applying Western minority stress frameworks in a collectivist context and propose responses grounded in trauma-informed care, LGBTQ-affirmative services, enumerated non-discrimination policy, and educator anti-stigma training.

**Conclusion:**

The psychological distress of this underserved population is structurally produced, psychologically mediated, and institutionally addressable. Affirmative mental health infrastructure, enumerated anti-discrimination policy, and LGBTQ-inclusive educator training can meaningfully reduce minority stress and its consequences.

## 1 Introduction

Gender identity and sexual orientation are established determinants of the risk of psychological distress. Across diverse national contexts, individuals identifying as lesbian, gay, bisexual, transgender, or queer (LGBTQ) exhibit significantly elevated rates of depression, anxiety, suicidal ideation, and post-traumatic stress disorder relative to heterosexual cisgender peers, with these disparities consistently attributable to minority stress rather than to inherent psychopathology (1, 2). In China, with an estimated LGBTQ population of 70 million by 2022, the confluence of historical pathologization, ongoing state restriction of LGBTQ civic space, and deeply embedded Confucian heteronormativity creates a high-stigma social environment with profound psychological implications (3). National survey data confirm that Chinese LGB college students exhibit depression prevalence of 48.1%, anxiety of 57.1%, and stress of 37.5%, rates substantially exceeding those reported for the general student populatio n. Direct comparison with heterosexual peers should be made with caution. Chinese studies of sexual minority and general student mental health differ in screening instruments, cut-off thresholds, and sampling frames, and no study to date has assessed sexual minority and heterosexual students within a single sampling frame using identical measures. What can be stated with confidence is the direction and approximate magnitude of the disparity rather than a precise difference in prevalence, and it is that disparity, not its exact size, from which the present study proceeds (5).

Unlike Western contexts where institutional non-discrimination policies and LGBTQ student organizations are more common, Chinese HEIs operate under a different policy environment: (a) there are no enumerated anti-discrimination protections based on sexual orientation or gender identity; (b) university counseling centers generally lack LGBTQ-affirmative training; (c) LGBTQ student organizations have been administratively dissolved in most institutions; and (d) state censorship of LGBTQ content in educational materials systematically erases LGBTQ lives from curricula.

Within this context, lesbian and bisexual women in Chinese higher education institutions (HEIs) occupy a particularly vulnerable position. Throughout this article, that phrase denotes cisgender women who identify as lesbian or bisexual or who describe themselves as same-sex attracted, which is the population from which this sample was drawn. Where the broader term LGBTQ is used, it refers to the wider population addressed by the literature or by policy, not to the participants in this study, whose accounts cannot be generalised to transgender, queer, or non-binary people. Research consistently demonstrates that LGBTQ students face worse educational, physical, and mental health outcomes than heterosexual peers, with schools playing a central role due to high rates of discrimination and victimization (6, 7). Gender-based and sexuality-based systems of stigma and discrimination do not operate independently but intersect to produce compound psychological distress that is qualitatively distinct from, and more severe than, either form of marginalization alone (8, 9). Yet this population has received disproportionately little psychiatric and psychological research attention. Existing Chinese LGBTQ mental health research has focused predominantly on gay and bisexual men, driven largely by HIV-related funding priorities, leaving the specific psychological mechanisms operating in lesbian and bisexual women’s educational experiences largely uncharted (4). The focus on HEIs as a research site is deliberate and theoretically motivated. Chinese universities are not merely incidental contexts for lesbian and bisexual women’s psychological distress: they are residential environments in which institutional cultures, peer dynamics, dormitory arrangements, and faculty relationships constitute the primary social world for the duration of study. Unlike workplaces or public spaces, HEIs shape identity development during a critical period of adult formation. State-controlled institutional cultures in Chinese HEIs have systematically closed LGBTQ student organizations, removed LGBTQ content from student media, and maintained heteronormative curricula — making the HEI an active structural producer of minority stress rather than a neutral background. Furthermore, lesbian and bisexual women in Chinese HEIs constitute a hidden population: there is no institutional record through which they can be enumerated, only approximately 5% of Chinese LGBTQ individuals disclose their identity in educational settings at school (57), and state censorship has progressively dismantled the digital community spaces through which participants might otherwise have been reached. The most psychologically vulnerable members of this population are simultaneously the most difficult to access, a research access paradox that this study acknowledges explicitly rather than minimising as a methodological inconvenience.

This study addresses that gap. Guided by Meyer’s (2) minority stress model and Hatzenbuehler’s (10) psychological mediation framework, supplemented by Crenshaw’s (8) intersectionality theory, we apply a qualitative reflexive thematic analysis design was applied to examine the structural and psychological dimensions of lesbian and bisexual women’s experiences in Chinese HEIs. Rather than documenting discrimination descriptively, the analysis traces the specific psychological pathways through which structural stigma, institutional inequity, and cultural heteronormativity become internalized as psychological distress. Two primary research questions are addressed.

1 How do same-sex attracted cisgender women in Chinese HEIs understand and experience the relationship between structural stigma and their own psychological well-being?
2 How do intersecting identity dimensions (including gender, sexual orientation, and regional background), shape the ways in which same-sex attracted cisgender women in Chinese HEIs experience and make sense of minority stress?

## 2 Theoretical frameworks

### 2.1 Minority stress theory

Meyer’s (2) minority stress model constitutes the primary psychological framework for this study. The model proposes that sexual minorities experience stressors unique to their minority status beyond those faced by the general population that accumulate to produce elevated rates of psychiatric disorder. Critically, the model distinguishes between distal stressors (objective, external events: discrimination, victimization, enacted stigma) and proximal stressors (subjective, internalized processes: internalized homophobia, expectation of rejection, identity concealment). Proximal stressors are theorized to mediate the relationship between distal stressors and self-reported psychological outcomes, meaning that external discrimination produces psychological harm primarily through activating internal processes of self-stigmatization and hypervigilance.

In the Chinese educational context, the minority stress framework identifies several key processes relevant to lesbian and bisexual women’s risk of psychological distress. Enacted stigma experienced through peer exclusion, teacher microaggressions, and institutional heteronormativity functions as a distal stressor activating the expectation of rejection — a state of chronic hypervigilance in which individuals scan social environments for potential threats. LGBTQ students in Chinese HEIs encounter unique institutional barriers, from exclusion in dormitory placement to the absence of affirming counselling services, and face higher rates of discrimination than their heterosexual peers (11, 12). This hypervigilance is cognitively demanding and emotionally depleting, producing rumination, impaired concentration, and reduced academic engagement even in the absence of active discrimination (10, 13). Internalized homophobia. The absorption of societal antigay stigma as negative self-regard constitutes the central proximal stressor, positively associated with depression, anxiety, suicidal ideation, and behavioral risk across multiple Chinese studies (5, 14, 15). Identity concealment, adopted as a protective response to anticipated rejection, carries its own psychological costs: inauthenticity in social relationships, chronic cognitive load from managing disclosure, and exclusion from the social support networks that moderate stress.

Crucially, Meyer’s framework also specifies protective factors buffering minority stress outcomes: community connectedness, positive LGBTQ identity development, and access to affirming social environments. These protective factors are structurally produced, they depend on institutional recognition of LGBTQ identities and on the availability of affirming peer and counselling communities. Their systematic absence in Chinese HEIs thus constitutes not merely a missed opportunity but an active structural condition that amplifies the risk of psychological distress by removing available buffers.

It is important to note, however, that minority stress theory was developed and validated primarily in Western, individualist cultural contexts with North American gay and bisexual male samples. Sun, Budge, et al. (16–18) have provided important empirical evidence for the model’s applicability in Chinese contexts, demonstrating its utility among Chinese gay and bisexual men and men who have sex with men; however, these studies have focused predominantly on male samples, leaving the model’s applicability to lesbian and bisexual women in China less thoroughly examined. Its direct applicability to a Confucian-influenced, collectivist context requires critical reflection. In collectivist cultures, the self is constituted relationally rather than individually (53): stigma experienced through family shame and filial obligation may produce psychological harm through mechanisms that Meyer’s original formulation does not fully specify. This study applies the minority stress framework with this cultural qualification: the framework provides conceptual scaffolding for understanding stigma-to-distress pathways, but interpretations are situated within the specific relational and institutional context of Chinese higher education.

### 2.2 Hatzenbuehler’s psychological mediation framework

Hatzenbuehler’s (10) psychological mediation framework extends the minority stress model by specifying three classes of psychological mechanism through which stigma produces psychological harm. First, social-cognitive processes: stigma activates ruminative cognition focused on stigma experiences and generates cognitive schemas of self-as-inferior or defective, schemas that directly impair mood, self-esteem, and motivation. Second, emotional regulatory processes: stigma exposure produces heightened shame and intensifies difficulties regulating negative affect, particularly following identity-invalidating social interactions such as microaggressions. Third, interpersonal processes: stigma disrupts social relationships and generates isolation, removing the interpersonal support that constitutes a primary buffer against stress-induced psychological deterioration. In the current study, all three pathways are operationalized through participants’ accounts, enabling the analysis to specify not merely that stigma causes harm but how it does so psychologically. The framework’s specification of mediating pathways is particularly valuable for a qualitative study because it enables interpretive analysis to move beyond phenomenological description toward mechanistic explanation, it connecting what participants experienced to why those experiences produce self-reported psychological outcomes.

### 2.3 Intersectionality theory

Crenshaw’s (8) intersectionality theory provides the structural-analytical complement to the psychological frameworks. The theory demonstrates that multiple systems of oppression sexism, heterosexism, classism, regionalism, do not simply add together but interact to produce qualitatively distinct forms of disadvantage for those positioned at their intersection. This framework has been applied productively in qualitative health research to illuminate how intersecting identities compound health disparities (19). For lesbian and bisexual women in Chinese HEIs, the interaction of gender-based and sexuality-based oppression generates a form of psychological vulnerability that exceeds what either axis of disadvantage alone would predict. The concept of intersectional invisibility (9) further specifies a psychological mechanism unique to multiply marginalized populations: individuals whose identities combine multiple subordinate characteristics are rendered invisible within both dominant frameworks and single-axis advocacy movements, producing additional psychological harm through non-recognition and the denial of affirming community membership. This study applies intersectionality not as a descriptive label but as an analytical tool directing attention toward the structural conditions that produce compound disadvantage, consistent with Moradi and Grzanka’s (20) call for structurally responsible intersectional research.

### 2.4 Stigma and discrimination against LGBTQ people in China: structures, forms, and psychological consequences

Stigma against LGBTQ people in China operates through multiple reinforcing structural levels, each with documented psychological consequences. Following Link and Phelan’s (21) foundational conceptualization, stigma is understood as the co-occurrence of labeling, stereotyping, status loss, and discrimination in a power context, a framework that illuminates how LGBTQ stigma in China is reproduced simultaneously at legislative, medical, cultural, and media levels. At the legislative level, same-sex relationships carry no legal recognition, denying LGBTQ couples family law protections and producing chronic legal precarity that constitutes a distal stressor (22). At the medical-institutional level, transgender presentations remain classified as mental disorders under China’s Classification and Diagnostic Criteria of Mental Disorders (CCMD-3), even as the ICD-11 has removed gender incongruence from its mental disorders section, a classification that directly reproduces pathologizing stigma in healthcare settings (23). At the cultural level, Confucian filial piety norms rooted in centuries of patriarchal tradition (24) frame non-procreative relationships as unfilial, producing pervasive familial stigma, including social coercion toward heterosexual marriage that Zheng et al. (25) have operationalized as a culture-specific stressor interacting multiplicatively with general minority stress to produce elevated self-reported symptoms of distress. These Confucian norms have historically constrained women’s educational and professional participation, with their effects still evident in contemporary China (26). At the media level, state censorship regulations prohibit LGBTQ content in television and online programming, systematically erasing LGBTQ lives from public visibility (22).

National survey data from Wang et al. (4), the first nationally representative assessment of anti-LGBTQ discrimination across China’s 31 provinces, have documented self-perceived discrimination across all measured institutional domains, with particularly severe discrimination in social service and legal settings. Discrimination levels were negatively correlated with regional economic development, a pattern consistent with research on educational resource inequality in China showing that decentralized financing has intensified geographic disparities in institutional quality and staffing (27, 28). In resource-poor regions, gender-based allocation of educational opportunities further disadvantages women (29), meaning that lesbian and bisexual women in peripheral institutions face simultaneously the material deprivation of underfunded educational environments and the most intense anti-LGBTQ stigma.

The psychological mechanisms through which structural stigma produces individual harm operate via Hatzenbuehler’s (10) three pathways. The social-cognitive pathway is activated by enacted stigma: discrimination experiences produce ruminative cognition (30), expectation of future rejection, and schemas of self-as-deviant that impair academic functioning and mood. The emotional regulatory pathway is activated by microaggressions and identity invalidation: even well-intentioned heteronormative responses by educators produce shame responses and heightened affect dysregulation in LGBTQ students (31). The interpersonal pathway is activated by social exclusion: peer rejection and dormitory anxiety generate chronic isolation that severs access to social support buffers (5). Evidence-based strategies for reducing stigma in educational settings, it including LGBTQ-inclusive curricula (12), enumerated non-discrimination policies (32), and LGBTQ-affirmative professional development for educators have demonstrated effectiveness in reducing victimization and improving mental health outcomes for LGBTQ students. Conversion therapy still practiced in China, with almost one in five transgender teenagers reported to have been forced into such practices constitutes an extreme institutional enactment of stigma with severe and documented psychological consequences including depression, anxiety, and suicidality (22).

## 3 Methods

### 3.1 Study design and epistemological position

A qualitative research design employing reflexive thematic analysis (33, 34) was adopted. Reflexive thematic analysis, in its contemporary formulation, explicitly positions the researcher as an active participant in knowledge construction rather than a neutral instrument for extracting pre-existing themes. This design aligns with the study’s epistemological commitment to critical realism: the position that there are real structural conditions stigma, institutional discrimination, resource inequalities that produce real psychological effects, but that participants’ accounts of these effects are always interpreted through subjective, culturally situated frameworks (46, 48). Critical realism supports the combination of structural analysis (identifying the mechanisms through which stigma produces harm) with interpretive analysis (attending to how participants make sense of those mechanisms), enabling the study to move between the structural and the phenomenological without collapsing one into the other.

This epistemological position has direct consequences for how analysis was conducted. In contrast to positivist qualitative approaches that treat researcher subjectivity as a source of bias to be minimized, the critical realist stance adopted here treats researcher positionality as analytically generative, the researcher’s interpretive framework, theoretical commitments, and awareness of their own social location shape which aspects of participants’ accounts become analytically salient. The implication is not that analysis is arbitrary but that it is situated, and that this situatedness must be made explicit and examined throughout the research process, not managed away through procedural controls (34).

### 3.2 Participants and sampling: sensitivity of the population and recruitment challenges

Recruiting lesbian and bisexual women in Chinese higher education presented substantial methodological, ethical, and practical challenges that are inseparable from the realities of this population’s situation and require detailed explanation. These challenges are not merely procedural difficulties to be noted and set aside; they are constitutive of the study’s epistemological and ethical character, and they directly shaped the size, composition, and interpretive limits of the sample.

#### 3.2.1 Why this population is particularly difficult to access

lesbian and bisexual women in Chinese HEIs represent a multiply hidden population. The concept of a “hidden population” in research methodology refers to groups whose members cannot be enumerated through conventional sampling frames because they lack visibility in public or institutional records (49). For lesbian and bisexual women in China, this invisibility is not incidental but structurally enforced through several intersecting mechanisms.

First, there is no legal recognition of LGBTQ identity in China. Same-sex relationships carry no legal status, there are no formal anti-discrimination protections based on sexual orientation or gender identity, and LGBTQ identities are absent from official university records, student registers, and administrative systems. This means there is no institutional infrastructure through which lesbian and bisexual women students could be systematically identified or contacted, in contrast to, for example, studies of students with disabilities or students from specific geographic backgrounds, where institutional records provide a sampling frame.

Second, disclosure of LGBTQ identity in China carries genuine and well-documented risks. Wang et al. (4) documented severe discrimination against LGBTQ individuals across family, educational, social service, and legal domains in a nationally representative survey. In the university context specifically, participants in this study and in prior research (11) described peer exclusion, microaggressions from educators, dormitory-based social isolation, and in some cases, family threats of forced heterosexual marriage as consequences of LGBTQ identity disclosure. Only approximately 5% of Chinese LGBTQ individuals disclose their identity at school, in the workplace, or in religious communities (57). The vast majority of lesbian and bisexual women students in Chinese HEIs are therefore not identifiable through any standard recruitment mechanism, because they have not disclosed their identity to anyone sample comprises cisgender lesbian and bisexual women within their institutional environment.

Third, state censorship of LGBTQ content under Xi Jinping’s administration has progressively dismantled the digital spaces through which LGBTQ Chinese individuals previously connected. University LGBTQ student organization WeChat accounts have been closed, LGBTQ-themed social media content is routinely removed, and the institutional LGBTQ organizations that existed on some campuses in the early 2010s have been administratively dissolved (22). The practical consequence for researchers is that the community infrastructure, student organizations, online groups, campus events through which LGBTQ participants might ordinarily be recruited no longer reliably exists at most Chinese institutions. This is not merely an inconvenience; it represents a structural restriction on research access that is itself a form of stigma enacted at the state level.

Fourth, the specific intersection of gender and sexual minority identity among lesbian and bisexual women creates compounded reluctance to participate. As documented throughout this study, lesbian and bisexual women in China face both the general LGBTQ stigma described above and the gender-specific pressure to conform to heterosexual marriage and reproductive expectations. Participating in research that required them to name and discuss their LGBTQ identity, to describe experiences of discrimination, and to trust a researcher with accounts that could identify them to family or institutional actors carried risks that were not trivial or hypothetical. For several participants, the decision to participate was described as carrying personal cost and requiring trust that was not easily extended.

#### 3.2.2 Recruitment strategy and its justification

Purposive sampling was employed to identify information-rich cases aligned with the study’s focus (35, 36). Inclusion criteria required: self-identification as lesbian or bisexual; cisgender gender identity; and current enrollment in a Chinese HEI. Eight participants were recruited from universities located in four province-level administrative regions: Beijing Municipality and the provinces of Zhejiang, Sichuan, and Fujian. The sample therefore spans one first-tier municipality (Beijing) and three provincial capitals (Hangzhou, Chengdu, Fuzhou), providing variation in regional economic development and in institutional type. Province-level region is used consistently throughout the article, since Beijing is a municipality rather than a province. This sample size, while modest, reflects the genuine difficulty of accessing this population rather than a limitation of research ambition. Each participant represented a successful navigation of considerable personal risk and institutional constraint to share their experience. Table 1 presents participant characteristics.

**Table 1:** Participant characteristics and self-reported psychological concerns.

No.	Pseudonym	Level	City (province-level region)	Major	Self-described identity	Reported psychological concern
1	Bai	Year 2	Chengdu (Sichuan)	Arts	Cisgender lesbian	Identity concealment; peer isolation
2	Shu	Year 4	Hangzhou (Zhejiang)	Fashion Design	Cisgender lesbian	Family rejection anxiety; self-silencing
3	Zeng	Year 3	Hangzhou (Zhejiang)	Arts	Cisgender bisexual	Enacted stigma; academic disengagement
4	Yan	Year 4	Chengdu (Sichuan)	Philosophy	Cisgender lesbian	Intersectional identity conflict
5	Ouyue	Year 4	Fuzhou (Fujian)	Linguistics	Cisgender lesbian	Single-axis attribution; internalized stigma
6	Hu	Year 3	Hangzhou (Zhejiang)	Arts	Cisgender lesbian	Dormitory anxiety; social exclusion
7	Peng	Postgrad	Beijing (municipality)	Communication	Cisgender bisexual	Microaggression exposure; rumination
8	Dei	Year 4	Beijing (municipality)	Social Work	Cisgender lesbian	Progress awareness; institutional advocacy

*N* = 8. Pseudonyms are used throughout. All participants self-identified using the descriptors listed. Reported psychological concerns are derived from participants’ own accounts during interview, not from standardised diagnostic instruments. Locations denote the province-level administrative region in which the participant’s institution is situated; institution names are withheld for participant protection. The concerns listed are the researcher’s summary of what participants themselves described and carry no diagnostic status.

#### 3.2.3 Implications for sample representativeness

The recruitment challenges described above have direct implications for the representativeness of the sample and must be stated explicitly rather than deferred to the limitations section. Purprosive sampling and snowball sampling through trust networks produces samples that are socially connected; participants who agreed to be interviewed are, by definition, connected to at least one other LGBTQ individual willing to make the introduction. This systematically excludes lesbian and bisexual women who are completely isolated those who have disclosed their identity to no one, who have no access to encrypted LGBTQ community spaces, and who experience their situation as too dangerous to discuss with anyone. These are likely to be among the most psychologically vulnerable members of the population. The accounts gathered in this study may therefore underestimate the severity of minority stress, internalized stigma, and psychological distress among the most hidden members of this population.

This limitation is not correctable within the current study design. It is a structural consequence of the conditions under which lesbian and bisexual women live in contemporary China: the same stigma and state restriction that create the psychological harms documented in this study also prevent the research access that would allow full documentation of those harms. This constitutes what might be called a research access paradox, the most vulnerable members of a stigmatized population are simultaneously the most important to reach and the most difficult to access, and it deserves explicit naming as a constraint on the knowledge this study can produce, not merely as a methodological imperfection.

### 3.3 Data collection

Semi-structured interviews lasting 60–90 min were conducted in Mandarin Chinese to minimize linguistic barriers. The interview protocol was developed specifically for this study (see Supplementary File 1 for the English-language version) and encompassed five theoretically grounded domains: (1) awareness and cognitive appraisal of gender inequality and stigma; (2) personal and academic experiences of discrimination and their affective and behavioral sequelae; (3) internalized stigma processes including shame, self-concealment, and identity conflict; (4) perceived institutional climate and its moderating effects on psychological well-being; and (5) coping strategies, help-seeking behaviour, and resilience resources. Protocol questions were piloted with two lesbian and bisexual individuals not included in the main sample and revised for clarity and cultural appropriateness. Interviews were audio-recorded with informed consent, transcribed verbatim, and translated into English by the primary researcher with bilingual review for accuracy. Translation followed a specified three-step procedure. The researcher who conducted the interviews produced the initial English rendering of each transcript. Translation was verified by the primary researcher (PhD in Education, University of Delaware, who works routinely in both languages). Where the two readings diverged, or where the meaning was culturally dense, the extract was back-translated into Chinese by a third bilingual reader who had not seen the original, and the two Chinese versions were compared (Supplementary file 2). Culturally specific terms were retained in transliteration with a gloss rather than replaced by an approximate English equivalent, because the semantic weight of expressions such as diu lian (loss of face), xiao (filial obligation), and bi hun (marriage evasion) is not carried by their nearest English counterparts. The Chinese originals of every extract reported in Section 4, paired with the English translations used, are provided in Supplementary File 2 so that readers with Mandarin can assess the rendering directly.

### 3.4 Data analysis

Reflexive thematic analysis was conducted following the six-phase approach described by Braun and Clarke (33) and elaborated in their subsequent methodological work (34, 37). This later work emphasizes that reflexive thematic analysis is not a method for “finding” pre-existing themes in data but for constructing themes through an iterative, researcher-involved interpretive process. The six phases, data familiarization, initial code generation, theme construction, theme review, theme definition, and write-up were followed non-linearly, with movement between phases as analytical understanding deepened.

Analysis proceeded in two integrated phases. In the first phase, inductive codes were generated from close reading of transcripts, attending to participants’ own language, emotional tone, and emphasis. In the second phase, coded material was systematically examined through the lenses of minority stress theory and Hatzenbuehler’s (10) mediation framework, identifying the specific psychological mechanisms operative within each emerging theme. This two-phase approach inductive coding followed by theoretically oriented interpretation, honors participants’ own framings while connecting emerging themes to the explanatory power of established psychological frameworks. This approach is consistent with Braun and Clarke’s (33, 34) reflexive thematic analysis, in which researcher theoretical commitments legitimately shape the interpretive process without predetermining the codes generated inductively from data. Reflexive thematic analysis was selected because the analytic aim was to construct patterns of shared meaning across the dataset rather than to produce idiographic accounts of individual meaning-making.

The route from raw data to final themes can be specified more precisely than a description of the six phases allows. Familiarisation produced margin notes and a summary memo for each of the eight transcripts. Coding generated 87 initial codes across the full dataset, of which 52 were applied to more than one transcript. These were clustered into 15 candidate themes. Review reduced the candidate set to the five themes reported here: 6 candidate themes were merged because their codes described a single underlying process at different levels of description, and 4 were reabsorbed into broader themes because they were carried by a single participant and could not be evidenced across the dataset. Definition and naming produced the theme statements used in Section 4. The complete coding tree, showing every initial code, the cluster it entered, and the candidate and final themes it fed, is provided as Supplementary File 3. Table 2 presents the same route in worked form for seven representative extracts and should be read as an illustration of the procedure described here rather than as a summary of the findings.

**Table 2:** Worked illustration of the analytic route from data extract to final theme.

Participant	Data extract	Initial codes	Psychological mechanism pathway	Theme generated
Hu	*“Gender inequality is all unequal treatments, expectations, and opportunities that some people go through just because of their gender…”*	Structural awareness; cognitive framing of disadvantage; involuntary vigilance	Social-cognitive: anticipatory anxiety	Theme 1: Awareness of structural inequality
Shu	*“LGBTQ women in my environment were more silent and afraid to express themselves.”*	Self-silencing; behavioral suppression; anticipatory concealment	Emotional regulatory: identity suppression	Theme 1: Awareness of structural inequality
Zeng	*“One male student told me once I tried a man I would not be lesbian anymore. It was humiliating…”*	Enacted stigma; invalidation; humiliation; academic interference	Interpersonal: peer enacted stigma	Theme 3: Enacted and internalized stigma
Ouyue	*“My supervisor asked me whether I was pretending to be a lesbian or whether it was just adolescent rebellious behaviour. I knew she was not trying to hurt me, but…”*	Intersectional invisibility; single-axis attribution; compound non-recognition	Interpersonal: exclusion from advocacy communities	Theme 4: Intersectional invisibility compounds psychological distress
Peng	*“No one incident was terrible enough to report. But together they made me feel that I did not belong. I started to wonder whether I was reading the situation wrong, whether I was too sensitive. That self-doubt was worse than any single comment.”*	Microaggression; rumination; academic cognitive interference	Emotional regulatory: affect dysregulation after microaggression	Theme 3: Enacted and internalized stigma
Dei	*“The LGBTQ student group gave me a space where I did not have to explain myself*	Community belonging; identity affirmation; stress buffering; academic re-engagement	Interpersonal: community connectedness as moderator	Theme 5: Attenuated outcomes where support is present
Ying	*“My parents keep sending me articles about conversion therapy. I feel like I cannot go home.”*	Familial stigma; coercive marriage pressure; relational rupture	Social-cognitive: filial guilt; shame activation	Theme 3: Enacted and internalized stigma

Data extracts are verbatim from translated transcripts; italics indicate direct quotation, except where a cell is marked as a summary of account. Initial codes were generated inductively in Phase 1 (33). Psychological mechanisms were assigned in Phase 2 through theoretical orientation toward Hatzenbuehler's (10) framework. The table is referenced in Section 3.4, where the coding and theme development procedure is described. Its purpose is to make one step of that procedure inspectable rather than to summarise the findings, which are reported in Sections 4.1 to 4.5. It is illustrative and not exhaustive: the complete coding tree for all initial codes is provided as Supplementary File 3.

Because the second analytical phase read inductive codes through minority stress theory and the psychological mediation framework, a procedure was required for codes that resisted or contradicted those frameworks. Three rules were applied. First, an inductive code was never rewritten to fit a theoretical category; where a code and a category did not correspond, the code was retained in the participant’s own terms and the mismatch recorded in an analytical memo. Second, discrepant codes were treated as findings rather than as noise, and were carried forward into the write-up. Third, where a discrepant code recurred across participants, it was used to qualify the framework rather than the framework used to reinterpret the participants.

Three instances show what this produced. Ouyue’s consistent attribution of her disadvantage to gender alone runs against the intersectional expectation that multiply marginalised individuals recognise compound disadvantage. Rather than coding this as an absence of critical awareness, the mismatch was retained and became the basis of Theme 4, in which self-invisibilising is analysed as an adaptive response to differential stigma. Dei’s account of incremental institutional progress and Bai’s account of a sustaining online community sit awkwardly with a framework organised around harm; neither was subordinated to the distress narrative, and both were built into Theme 5, which reports the conditions under which minority stress outcomes are attenuated. Shu’s statement that her family would more readily accept a gay son than a lesbian daughter contradicts the assumption that lineage obligation in the Confucian family bears most heavily on sons. That contradiction was not resolved analytically; it is carried into the Discussion, where it is used to specify how marriage pressure is gendered.

Analytical memos were maintained throughout, documenting interpretive decisions, reflexive observations, and emerging theoretical connections. Cross-case synthesis identified shared patterns while preserving individual variation. Trustworthiness was addressed using (52) four criteria. Credibility was strengthened through member-checking: three participants reviewed summary findings and confirmed their accuracy, and prolonged engagement with the data across multiple analytical phases allowed interpretive claims to be tested against the full corpus rather than isolated extracts. Transferability is supported through the thick description of participant context, structural conditions, and the specific HEI setting provided in Sections 3.2 and 2.4, enabling readers to assess applicability to comparable contexts. Dependability is addressed through the analytical memo trail, which documents the reasoning behind interpretive decisions at each phase and creates an auditable record of how themes were constructed. Confirmability is addressed through the reflexive bracketing practices described in Section 3.5, in which the researcher’s theoretical commitments were examined at each analytical phase to make explicit how they shaped emerging interpretations. The concept of data saturation, as understood in grounded theory or thematic saturation approaches, does not apply in the same way within reflexive thematic analysis (34): the adequacy of the sample is justified by the richness and analytical depth of the material generated rather than by the cessation of new codes. Eight participants across four-province level regions, representing lesbian and bisexual and geographic contexts, produced sufficient material to construct five well-evidenced and analytically distinct themes. Reflexivity was operationalized not only as an ethical disclosure in Section 3.5 but as an ongoing analytical practice: at each phase of analysis, the researcher’s positionality was examined in relation to the emerging interpretations, asking whose perspective was being centered and what the researcher’s own social location might render invisible or over-salient.

To ensure transparency and auditability of the analytical process, the full hierarchical coding tree, it illustrating the progression from initial inductive codes to candidate themes and final refined themes, is provided as Supplementary File 3.

### 3.5 Researcher positionality and reflexivity

The primary researcher does not identify as LGBTQ. However, the researcher is Chinese and culturally embedded in the context under study. This positionality creates a specific asymmetry: insider status in relation to Chinese cultural norms, outsider status in relation to the LGBTQ experience. This was managed through reflexive practices described below. This positionality creates an asymmetry in the research relationship that was not manageable through procedural safeguards alone. A significant methodological gap in this original study design was the absence of consultation with an LGBTQ-specialist colleague or advisory body prior to finalising the research design. Such consultation, as recommended by Allen (38) and Warner (39), would have provided an external check, at the design stage, on the specification of the target population and on the inclusion criteria applied to it. Researcher reflection, while necessary, is not sufficient: it benefits from external challenge by those with lived or specialist knowledge of the community under study. This is now acknowledged as a methodological limitation and as a practical recommendation for future research involving LGBTQ participants in high-stigma contexts. Three further reflexive considerations shaped the analysis and are disclosed here as analytically relevant rather than merely ethically required.

First, the researcher’s outsider status in relation to LGBTQ experience meant that the theoretical frameworks of the minority stress theory and intersectionality, served not only as analytical lenses but as necessary bridges, providing conceptual scaffolding for understanding experiences that the researcher could not draw on personal knowledge to interpret. This increased reliance on theoretical frameworks carries the risk of over-systematizing participants’ accounts into pre-specified categories. This risk was managed through the inductive first phase of coding and through sustained attention in analytical memos to moments where participants’ accounts resisted or complicated theoretical expectations.

Second, the researcher’s insider status in relation to Chinese cultural context meant that Confucian norms around filial piety, family obligation, and marriage pressure were familiar as lived social expectations rather than only as analytic categories. That familiarity carries its own interpretive risk, since what is taken for granted can go unexamined, and norms that a cultural outsider would notice and interrogate may pass without comment. The use of Mandarin-language interviews, bilingual translation review, and member-checking partially addresses this gap, but the interpretations offered in this paper should be read with awareness of this limitation.

Third, the power asymmetry between a researcher with institutional affiliation and participants who are members of a stigmatized and legally unprotected population in China was acknowledged throughout. Interview questions were designed to avoid pressing participants on aspects of their identity they had not volunteered, and participants were explicitly reminded of their right to withdraw or decline questions at any point.

### 3.6 Ethical considerations

This study was conducted in full accordance with the Declaration of Helsinki (58) and the ethical standards of the relevant national and institutional research committees. Ethical approval was granted by Institutional Review Board of Xi’an Eurasia University, approval reference number XEU-03201-2025, prior to data collection commencing. All participants provided written informed consent prior to interview. Participants were fully informed of the study’s purpose, their right to withdraw at any point without consequence, the confidentiality of their data, and the specific protections implemented given the social and legal risks of LGBTQ identity disclosure in China. These protections included pseudonymisation of all identifying information prior to analysis; encrypted storage of all data on password-protected devices; removal of all geographic identifiers specific enough to identify participants’ institutions; and explicit assurance to all participants that the research findings and data would not be shared with any Chinese governmental or institutional authority. Participants were provided with mental health resource contacts in their respective province-level regions at the conclusion of their interview. What those resources could offer deserves direct comment, since it bears on the phenomenon the study documents. Provincial and university counselling services in China are not, as a rule, LGBTQ-affirmative. None of the university counselling centres in the participants’ regions advertises affirmative practice, staff are not required to receive training in sexual minority mental health, and confidentiality in relation to academic advisers and to families is not guaranteed in the way it would be in many other jurisdictions. Participants were therefore given a two-tier list: general mental health and crisis services, together with the small number of community-based sexual minority support organisations and helplines operating and reachable at the time of data collection. These included community-based helplines and online support groups that were verified as operational at the time of data collection; specific names are withheld to protect the organisations from potential scrutiny. The narrowness of the available referral options is itself part of the institutional deprivation reported in Section 4.2.

## 4 Results

**Figure 1:**
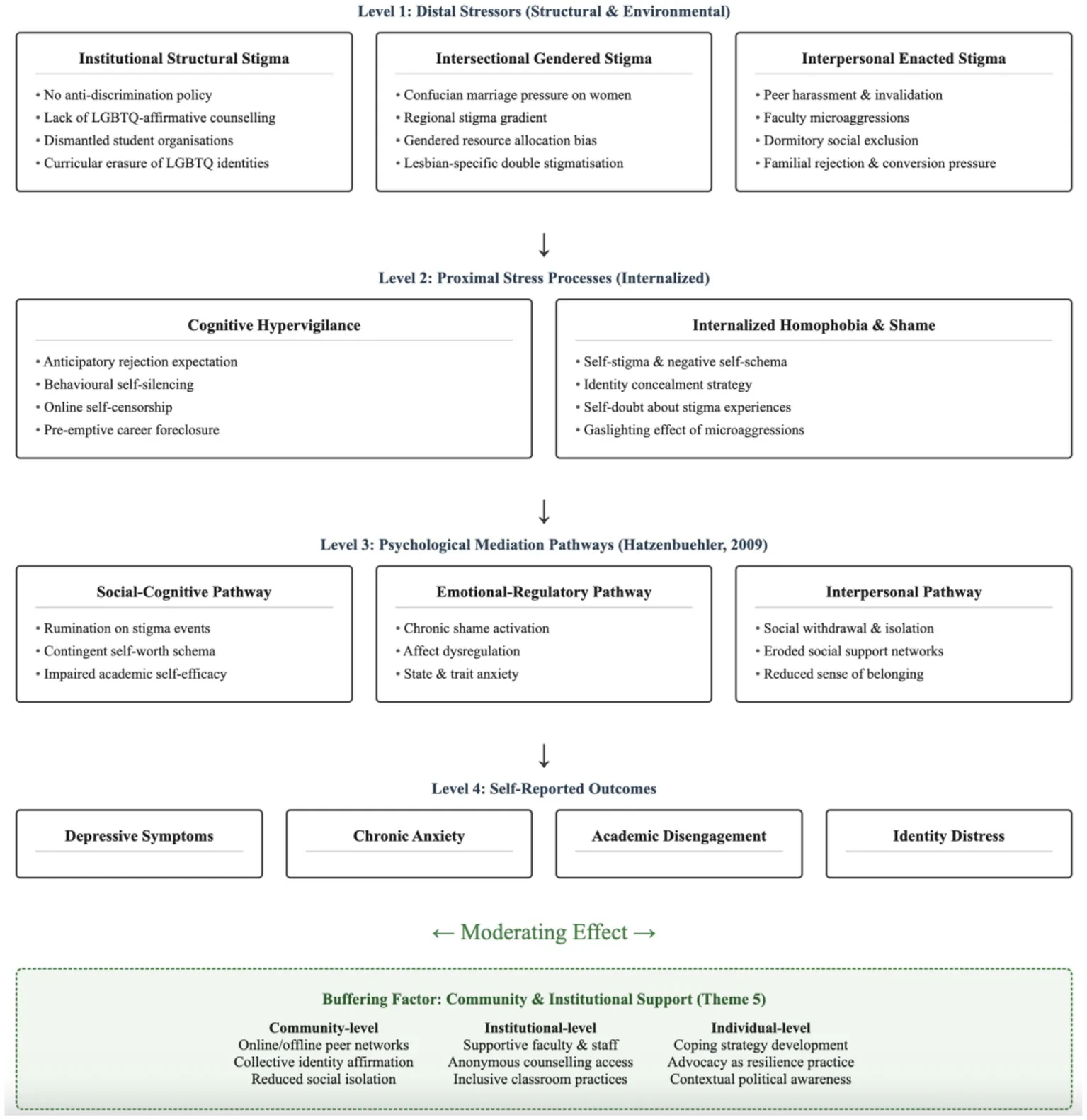
Conceptual framework: Minority stress mediation model. The model integrates Meyer's (2) minority stress theory, Hatzenbuehler's (10) psychological mediation framework, and Crenshaw's (8) intersectionality theory.

Five themes were constructed through reflexive thematic analysis, each representing a distinct psychological domain of lesbian and bisexual women’s experience in Chinese HEIs. Table 3 presents the cross-case synthesis of key features across themes, Figure 1 presents the conceptual model relating the five themes to one another, and Table 2 illustrates the analytic route from data extract to theme for a representative subset of the corpus. Figure 1 summarises the argument developed across Sections 4.1 to 4.5 and may be read either before or after them. The themes are presented not as “findings” that exist independently of the researcher’s interpretive work but as co-constructions: the product of the interaction between participants’ accounts and the theoretical frameworks and analytical commitments the researcher brought to the data (34).

**Table 3:** Cross-case features underpinning the five thematic domains.

Theme	Minority stress process	Psychological mechanism (10)	Self-reported outcome	Participants reporting
1. Awareness of structural inequality	Distal stressor: enacted stigma/expectation of rejection	Social-cognitive: anticipatory anxiety; cognitive vigilance; rumination	Self-reported chronic anxiety; academic disengagement	All 8
2. Institutional resource deprivation	Distal stressor: structural discrimination	Social-cognitive: contingent self-worth impairment; belonging threat	Reduced motivation; self-reported depressive symptoms	7/8
3. Enacted and internalized stigma	Distal → proximal: internalized homophobia; shame	All three pathways: social-cognitive, emotional regulatory, interpersonal	Self-reported depressive symptoms; shame; identity distress; social withdrawal	All 8
4. Intersectional invisibility compounds psychological distress	Proximal: stacked stress; intersectional invisibility	Compound identity conflict; self-invisibilizing as adaptive defense	Compound psychological distress; exclusion from resilience resources	7/8
5. Attenuated outcomes where community and institutional support are present	Moderator: social support; community belonging; perceived institutional safety	Community connectedness moderating minority stress pathway	Attenuated outcomes where present; amplified where absent	All 8

Minority stress processes follow Meyer's (2) taxonomy: (distal stressors/objective external events; proximal stressors/subjective, internalized processes.) psychological mechanisms follow Hatzenbuehler's (10) tripartite framework. Participant counts reflect the number of participants whose accounts provided evidence for the theme.

A terminological note governs everything that follows. No diagnostic instrument was administered in this study, no participant was clinically assessed, and no clinical judgement is offered about any participant. Where the analysis refers to anxiety, depressive symptoms, shame, rumination, or distress, these terms denote psychological states that participants described in their own words and that the researcher has interpreted through the study’s theoretical frameworks. They are inferences from interview accounts, not diagnoses, and the strength of the evidence for them is the strength of a qualitative account rather than of a validated measure. Statements about prevalence in the wider population are attributed to the survey literature and are distinguished throughout from what this sample can support.

Conceptual Framework of the Minority Stress Mediation Model (Figure 1) from structural stigma to self-reported psychological outcomes among lesbian and bisexual women in Chinese higher education. Distal structural conditions activate proximal stress processes, which operate through the three mediating pathways specified by Hatzenbuehler (10) to produce the outcomes participants described. Social and institutional support moderates the pathway at the point indicated, attenuating outcomes where it is present and amplifying them where it is absent. Numbers in the figure indicate the theme in Section 4 in which each element is evidenced.

To illustrate the overall pathway from structural stigma to psychological outcomes among participants, a conceptual model derived from the thematic analysis is presented in Figure 1. The model maps the five identified themes onto the minority stress framework and the three psychological mediation pathways proposed by Hatzenbuehler (10), with the buffering effect of support marked separately.

### 4.1 Theme 1: awareness of structural inequality as anticipatory anxiety and cognitive vigilance

All eight participants demonstrated an awareness of gender inequality that was not merely cognitive but affectively charged experienced as a chronic background state of alertness and anticipation of harm. This pattern is consistent with Meyer’s (2) operationalization of the expectation of rejection as a proximal stressor: awareness of one’s stigmatized status activates a vigilance state in which social environments are continuously scanned for potential threats, a process that is cognitively depleting and anxiety-generating even in the absence of active discrimination. Hollinsaid et al.’s (13) longitudinal study provides empirical support for this mechanism: hypervigilance was identified as a significant mediator between stigma exposure and internalizing psychopathology in sexual minority young adults, establishing that the vigilance state generated by awareness of stigma constitutes a significant risk factor for psychological distress in its own right.

Hu’s account illustrates the cognitive appraisal on which this vigilance rests:

"Gender inequality is all unequal treatments, expectations, and opportunities that some people go through just because of their gender. It is a situation whereby these people are treated differently on the basis they are male, female, or even LGBTQ, making them a disadvantaged group."

From the researcher’s interpretive position, what is analytically significant in Hu’s account is not simply the content of her definition but its origin: the precision and comprehensiveness of her description reflects a person who has been required to understand her social position in order to navigate a stigmatizing environment, not because she sought intellectual engagement with the topic by choice. The extract evidences the appraisal component of this theme, which is the accurate reading of a stigmatising environment, and it is presented on that basis alone. It does not by itself evidence hypervigilance, which is an attentional and behavioral state rather than a cognitive appraisal, and no such claim is made from it. What Hu’s appraisal establishes is that the vigilance documented in the extracts that follow is a response to conditions participants read correctly rather than a distortion of them. Adaptive hypervigilance generates anxiety through the same mechanism that makes it protective. The psychological cost is cumulative — sustained vigilance depletes attentional and emotional resources, impairing concentration, interpersonal functioning, and sleep (10, 13).

The vigilance itself is visible in what participants did rather than in what they knew. Shu described its behavioral expression:

“LGBTQ women in my environment were more silent and afraid to express themselves.”

Self-silencing is not a personality trait. It is a minority stress adaptation. It reduces the risk of enacting a stigmatized identity in public settings. But it carries its own psychological cost. The inauthenticity required to maintain suppression constitutes a proximal stressor with cumulative effects (2). The chronic cognitive effort of self-monitoring is exhausting. It depletes the executive functioning resources needed for academic engagement.

Peng introduced a dimension absent from most minority stress accounts: the psychological cost of digital surveillance and online censorship.

“I used to find community through LGBTQ groups on WeChat. When those accounts were closed, I lost a space where I could be myself. Now I am more careful about what I write or share online. That carefulness is exhausting.”

The loss of digital community is not merely an inconvenience. It severs the interpersonal pathway of support that moderates minority stress outcomes. Peng’s account also supplies the attentional dimension that Hu’s appraisal does not. Being more careful about what she writes and finding that carefulness exhausting describes continuous self-monitoring of her own output, sustained across contexts and without a defined endpoint, which is what hypervigilance consists of at the level of attention rather than belief. The cognitive effort of digital self-censorship is a persistent drain on attentional resources.

### 4.2 Theme 2: institutional resource deprivation as a proximal stressor undermining academic self-efficacy

Seven of eight participants described systematic disparities in access to educational resources, leadership opportunities, and research funding that carried psychological consequences extending beyond their material effects. These accounts document the social-cognitive mechanism identified by Hatzenbuehler (10): structural exclusion activates cognitive schemas of inferiority and unworthiness that impair academic self-efficacy and reduce engagement independent of any specific discriminatory act. The construct activated here contingent self-worth (47) describes a state in which self-evaluation becomes dependent on external validation that the institution consistently withholds, producing chronic low-grade depression and motivational impairment.

Ying described the resource allocation dynamics and their affective consequences:

“Male students are given more opportunities such as leadership roles, and they are even given larger shares during academic trips’ funding and research funding. These actions usually discourage female and LGBTQ students from participating in such activities.”

The word “discourage” in Ying’s account is doing significant psychological work. It names not merely an external barrier but an internal state produced by that barrier. From a reflexive analytical position, it is worth noting that Ying presents this as observable institutional fact before she names its psychological consequence suggesting that for her, the structural and the psychological are not separate domains but aspects of a single lived reality. This is consistent with Crenshaw’s (8) argument that structural and psychological analyses must be integrated rather than sequenced.

Hu’s account introduced the regional dimension of this effect, revealing how geographic context modulates the risk of psychological distress:

“There is no difference in the skewing of educational resources for gender in cities, but educational resources in poorer areas are skewed in favour of males, and there is limited tolerance of LGBTQ women — those women are described as sick.”

The characterization of lesbian and bisexual women as “sick” in resource-poor regional contexts is not mere social intolerance but institutional enacted stigma with specific psychological consequences, it reproduces the pre-2001 pathologization framework in environments where educational resources, including access to affirming information and mental health support are scarcest. Wang et al.’s (4) national survey confirms that discrimination severity in China is inversely correlated with regional economic development. For lesbian and bisexual women in peripheral institutions, this produces what Zheng et al. (25) term stacked stress: the simultaneous experience of material resource deprivation and intensified psychological stigma whose combined psychological impact exceeds additive prediction.

Ying extended this account by reflecting on how resource exclusion affected her academic future:

“I stopped applying for research positions in my second year. I did not think I had a realistic chance, once I admitted my sexuality. My identity made me invisible to the faculty who controlled those opportunities.”

Zeng connected exclusion of this kind directly to academic outcome: “LGBTQ is a group in the community that faces high discrimination since they are considered to be immoral, and this can affect even their academic performances.” The analytic value of the extract is that Zeng identifies the pathway herself. She is describing the cognitive interference effect that Hatzenbuehler’s (10) framework specifies, in which attention and effort are diverted from academic work toward the management of stigma. Her account is treated here as data to be interpreted rather than as testimony to be accepted at face value, but the convergence between what she describes and what the framework predicts is analytically notable, and it is reported in this theme rather than in Theme 1 because what it describes is an academic consequence rather than an anticipatory state.

This represents a psychological consequence that is rarely named directly in minority stress research, anticipatory career foreclosure. The expectation of discrimination in future professional contexts leads to pre-emptive withdrawal from opportunities. The psychological cost operates through the social-cognitive pathway. Self-schemas of being unworthy or invisible generate not only present-tense depressive symptoms but also a constriction of imagined futures.

### 4.3 Theme 3: enacted and internalized stigma and their psychological consequences

This theme documents the full minority stress sequence across all three of Hatzenbuehler’s (10) mediation pathways. Its central organising concept is the absorption of structural condemnation into self-concept: the mechanism by which enacted stigma (external discrimination) crosses the boundary into internalized stigma (self-directed shame and self-invalidation), and through which each successive layer of externally produced harm generates its own internally sustained psychological cost. The shame, identity concealment, and depressive symptoms documented here are not parallel phenomena catalogued together for convenience; they are causally connected expressions of a single underlying process. It is the most densely evidenced theme.

#### 4.3.1 Social-cognitive pathway: internalized shame and identity distress

Ying’s account provides the clearest documentation of the social-cognitive pathway:

“My parents keep sending me articles about conversion therapy, as they think this is a psychological problem. I feel like I cannot go home. Sometimes I would not want to have any conversation with my parents. I am afraid to talk with them now — they always want to educate me.”

The analytic weight of this account operates on two levels simultaneously. At the first level, the repeated sending of conversion therapy materials constitutes a form of familial enacted stigma that communicates consistently and from the most intimate relational context, that Ying’s identity is disordered and requires correction. Meyer's (2) minority stress model identifies this mechanism precisely: when stigmatizing messages originate from primary attachment figures rather than strangers or institutions, their absorption into self-concept is deeper and more resistant to challenge. At the second level, Ying’s progressive withdrawal from parental communication from not wanting conversation to being afraid to speak, documents the interpersonal psychological cost of this enacted familial stigma in real time. The relationship that should constitute the primary source of relational safety has become a source of chronic identity invalidation and anticipatory threat. This is consistent with descriptions of internalized homophobia as conceptualized by Meyer (2) and documented among Chinese LGBTQ populations by Xu et al. (15) and Wen and Zheng (14): the external stigmatizing message is absorbed inward, producing shame, relational rupture, and the subjective experience of one’s own identity as something requiring concealment even from those closest. In the terms of the present analysis, this is associated with self-reported low mood, shame-based affect dysregulation, and interpersonal withdrawal with, all three of Hatzenbuehler's (10) mediation pathways activated through a single family relationship.

#### 4.3.2 Emotional regulatory pathway: microaggressions and affect dysregulation

Ouyue stated:

"My supervisor asked me whether I was pretending to be a lesbian or whether it was just adolescent rebellious behaviour. I knew she was not trying to hurt me, but…"

The psychological significance of this interaction is not diminished by the teacher’s lack of hostile intent. Microaggressions defined as brief, often unconscious exchanges communicating derogatory messages to stigmatized group members, exert cumulative psychological effects through the emotional regulatory pathway: each identity-invalidating interaction activates heightened shame, producing affect dysregulation that impairs subsequent emotional functioning (10, 31). Framings that position a participant’s identity as inauthentic or developmentally immature invalidate identity coherence and constitute a clinically significant stressor even when no hostile intent is present. Where a participant’s account trails off or breaks during interview, this is analytically significant: it marks the point at which affect exceeded verbal articulation, suggesting that the emotional regulatory cost of the original encounter remained active at the time of interview rather than merely recalled as a historical event. This is consistent with research documenting that microaggression exposure has sustained effects on emotional functioning that outlast the immediate interaction (31).

Peng described the cumulative weight of repeated low-level invalidation:

“No one incident was terrible enough to report. But together they made me feel that I did not belong. I started to wonder whether I was reading the situation wrong, whether I was too sensitive. That self-doubt was worse than any single comment.”

Peng’s account introduces a secondary psychological mechanism: the gaslighting effect of microaggression accumulation. Each individual incident falls below the threshold for formal complaint. Collectively, they produce a chronic state of identity invalidation. The self-doubt Peng describes ‘whether I was too sensitive’, which reflects the internalization of the institutional message that her experiences are not legitimate. This secondary internalization constitutes an additional proximal stressor layered onto the original microaggression exposure.

#### 4.3.3 Interpersonal pathway: social exclusion, isolation, and psychological distress

Zeng’s account documents the peer form of this pathway:

“One male student told me ‘once I tried a man, I would not be lesbian anymore’. It was humiliating. Also, people blamed HIV and other bad things on us, because they agreed that we had disrupted social harmony.”

Two mechanisms operate in this extract. The first is invalidation: her identity is construed as a condition awaiting correction rather than as a self-description to be taken seriously, and the correction proposed is itself a sexual demand. The second is contamination, in which she is assigned collective responsibility both for a public health threat and for a breach of social harmony. Both are interpersonal in Hatzenbuehler’s (10) sense. They operate by making ordinary peer contact a site of anticipated humiliation, and their effect is to reduce the peer relationships through which support would otherwise become available.

Hu’s account of dormitory anxiety and classroom exclusion documents the interpersonal pathway with particular clarity:

"Straight women are sometimes, worried or afraid of us if we live in the same dormitory, and male students think our behaviour is not normal. In the classroom, they use this perception to isolate us. In group assignments, I usually go to the group that is not full, I chose to join lately the group, it seems more acceptable."

The dormitory context is particularly significant given the residential character of Chinese university life: unlike daytime educational environments, dormitory exclusion offers no respite and no alternative social context, constituting a total-environment stressor. Unlike daytime contexts, where LGBTQ students can seek alternative spaces, dormitory exclusion constitutes a total-environment stressor. The psychological implications of chronic peer exclusion in a residential educational setting are severe. The interpersonal pathway specified by Hatzenbuehler (10) demonstrates that stigma-driven isolation severs access to social support the primary stress buffer in the minority stress model. Zhou et al. (40) document that social exclusion among LGB young adults in China produces a self-reinforcing cycle of distress: stigma generates withdrawal, withdrawal deepens isolation, isolation intensifies internalized stigma, and internalized stigma worsens mood.

Shu’s account of gendered family stigma documents the culture-specific stressor of marriage pressure:

"I do not know how to tell my relatives and parents, they could be really angered or confused, and may force me to act like others, like marry a man at the right age (after graduation). But for my little brother, they accepted that he is gay."

This differential family response, accepting a gay son while threatening forced heterosexual marriage for a lesbian daughter illustrates the gendered character of Confucian heteronormativity and its specific psychological implications for lesbian and bisexual women. Zheng et al. (25) operationalized this marriage pressure as a culture-specific stressor with three measurable dimensions (parental, social, and internalized pressure), finding that it interacted multiplicatively with general minority stress to produce the most severe self-reported psychological outcomes. The psychological mechanism is values conflict an unresolvable tension between authentic sexual identity and culturally sanctioned filial duty that constitutes a chronic proximal stressor producing depressive rumination and interpersonal withdrawal. Importantly, this stressor is distinctly gendered: Confucian reproductive obligation is more rigidly enforced for women than for men, meaning lesbian and bisexual women face a culture-specific stressor of greater intensity than that faced by gay men,a pattern that existing minority stress frameworks, developed primarily with male samples, do not fully capture.

### 4.4 Theme 4: intersectional invisibility compounds psychological distress

Seven of eight participants explicitly identified intersecting identities as central to their experience of disadvantage. Bai articulated the distinctiveness of her intersectional position:

"Being a queer woman affects how others perceive me. It’s not just about being a woman; it’s about how my sexual orientation intersects with that, leading to unique experiences."

The concept of intersectional invisibility (9) provides the psychological mechanism for understanding Ouyue, who framed her disadvantage primarily as a general gender issue. From a reflexive analytical position, it is important not to simply correct Ouyue’s framing as analytically insufficient, her single-axis attribution may reflect her genuine experience of disadvantage, in which gender-based barriers are more visible and more immediately pressing than sexuality-based ones. The intersectional invisibility framework proposes that this is not a misperception but an adaptive defense attributing disadvantage to the less stigmatized category of gender protects against the additional psychological cost of LGBTQ disclosure. But this defense simultaneously forecloses access to the LGBTQ community resources that constitute a primary resilience mechanism. The psychological trade-off is thus, short-term protection from stigma purchased at the cost of longer-term exclusion from affirming support.

The cumulative minority stress experienced by participants across family, peer, institutional, and public discourse domains represents what Zheng et al. (25) term stacked stress: the simultaneous experience of distal stressors (discrimination, enacted stigma, bullying) and proximal stressors (internalized homophobia, concealment, marriage pressure, anticipatory rejection) interacting multiplicatively to produce self-reported psychological outcomes more severe than additive models predict. For lesbian and bisexual women specifically, this stacked stress carries an additional gendered dimension that existing minority stress frameworks, developed in Western individualist contexts with primarily male samples, do not fully specify, a theoretical gap this study identifies as a priority for future development.

Dei offered a more structurally sophisticated account, one that reflects the analytical depth that sustained engagement with one’s own position can produce:

“I can see that things are slowly changing. There are more students who are aware of LGBTQ issues now than there were when I started. But the institutions have not caught up. The awareness is there but it is not protected.”

This distinction between individual-level awareness and institutional protection is analytically important. Dei is identifying the mechanism through which structural conditions maintain the risk of psychological distress even as cultural attitudes shift. Individual awareness of LGBTQ existence does not reduce minority stress if it is not institutionally protected. The expectation of rejection, a core proximal stressor, operates at the institutional level. Individual attitudinal change does not deactivate it. This finding has direct implications for intervention design: institutional reform cannot be substituted by cultural awareness campaigns.

### 4.5 Theme 5: attenuated outcomes where community and institutional support are present

Participants demonstrated both the psychological costs of inadequate institutional support and the resilience processes available when affirming conditions exist. Shu articulated a contextually sophisticated understanding of protective community belonging:

"Schools can acquiesce to the existence of sexual minority clubs or promote them as official endorsements with soft advocacy. Being vocal is an effective way for people who have been treated unfairly to speak out about their experiences so that more people realise there is a problem."

Shu’s framing of advocacy as “soft” is analytically significant in the Chinese political context: she is not simply expressing a preference for gradual change but identifying the specific political opportunity structure within which LGBTQ advocacy is viable without triggering institutional repression. This political-contextual awareness shapes the form of resilience available to participants and is itself a psychological resource, the ability to identify feasible pathways for change moderates the helplessness that characterizes the most damaging forms of minority stress (2).

Li et al. (5) demonstrated that social support significantly moderated the relationship between school bullying and internalized homophobia among Chinese LGB students when social support was available, the pathway from distal stigma to proximal psychological harm was substantially attenuated.

Participants consistently identified educator professional development as a structural lever for reducing microaggression exposure. Bai, whose experience of community belonging operated primarily through hidden digital networks rather than any institutionally recognised space, framed the professional development need not in terms of policy reform but in terms of the daily cost of enforced invisibility:

“Sometimes I think the problem is not that teachers do not know we exist. They do. The problem is that knowing we exist and acknowledging us officially are two very different things. In China, that gap, between knowing and saying, is where we live. If teachers were trained to at least close that gap in the classroom, even a little, it would mean we did not have to keep pretending that we do not belong to each other. Right now, we find each other quietly, carefully. A training programme would not change the law. But it might make it slightly less dangerous to be seen.”

Bai described a coping mechanism that reveals how digital spaces function as psychological refuges when physical institutional spaces are hostile:

“I found an online community where I could talk about my identity without fear. It was a small group. We were careful about what platform we used. It helped me feel less alone.”

From a psychological intervention standpoint, this recommendation targets the structural source of emotional regulatory pathway activation: the absence of LGBTQ-affirming knowledge in educator preparation that produces identity-invalidating interactions not from hostility but from ignorance. Kosciw et al. (41) and Seelman et al. (42) confirm that perceived staff supportiveness significantly reduces both LGBTQ victimization exposure and mental health symptom burden. In the Chinese context, professional development represents a particularly high-leverage intervention precisely because most educator microaggressions arise from structural knowledge gaps rather than deliberate prejudice and knowledge gaps are addressable through training without requiring the broad societal attitude change that is politically foreclosed in the current Chinese governance context.

## 5 Discussion

This study provides a psychologically grounded account of how lesbian and bisexual women’s marginalization in Chinese HEIs is produced, mediated, and potentially addressed. The findings extend the minority stress literature to this underexamined population in a high-stigma, collectivist context, while identifying clear targets for psychological and institutional intervention. Three substantive discussions follow.

### 5.1 The psychological architecture of minority stress: extending and qualifying the Western framework

The full minority stress sequence, running from the distal stressor → to proximal stressor → to self-reported psychological outcome, is clearly operationalized across all five themes. Enacted stigma (Zeng’s public humiliation, Peng’s faculty microaggression, Hu’s classroom exclusion) functions as the distal stressor activating proximal processes: the expectation of rejection (Shu’s chronic self-silencing), internalized homophobia, and identity concealment. These proximal processes produce documented self-reported psychological outcomes: chronic anxiety through hypervigilance, depression through shame and self-stigma, and social withdrawal through the interpersonal pathway. This psychological architecture is consistent with findings from institutional settings documented by Meneguzzo et al. (43) among TGD individuals, where rigid cisnormative frameworks similarly activated the same three mediation pathways, producing confusion, psychological strain, and disrupted identity coherence.

Crucially, however, the Chinese context adds two stressors not fully captured in Western minority stress research: the culture-specific stressor of marriage pressure (25) and the regional gradient of stigma severity (4). Research confirms that Chinese attitudes toward homosexuality are significantly shaped by gender equality awareness, with adherence to traditional gender-role beliefs amplifying negative evaluations of homosexuality at the individual level. Regional variation is documented separately, as Wang et al. (4) found discrimination severity inversely correlated with regional economic development. The Confucian gendering of reproductive obligation, which historically confined women to domestic roles while men pursued professional careers (24, 26), distributes the marriage pressure stressor differently for women and for men rather than simply more heavily on one. Sun et al.’ work with Chinese gay and bisexual men has documented how cultural modifiers — including family obligation and social visibility norms — shape the minority stress pathway in ways not fully captured by the original Western model (16–18). The present findings extend this line of inquiry to women, identifying gendered stressors — specifically, Confucian marriage pressure — that are absent from male-sample research yet carry severe psychological consequences. Role attitudes in contemporary Chinese society carry measurable socio-psychological consequences, a finding that supports the case for cultural adaptation of frameworks developed elsewhere. This finding identifies a specific gap in the minority stress literature requiring theoretical elaboration for adequate application to non-Western, non-male populations.

That claim requires specification, since lesbian women and gay men violate the same heteronormative expectation and the asymmetry is therefore not self-evident. Three differences emerged in participants’ accounts. The first is timing. The socially sanctioned window for women’s marriage is narrower and earlier, so pressure arrives sooner and escalates faster, and the public category of the unmarried older woman carries a stigma for which there is no male equivalent of comparable force. The second is the content of the obligation. A son’s failure to marry tends to be construed as a deferred failure of lineage continuation, whereas a daughter’s tends to be construed as an immediate failure of family standing, visible to relatives and neighbours, which locates the pressure in the everyday relational field rather than in an abstract duty. The third is the availability of exit. Economic independence, geographic distance, and the tacit accommodation of an unmarried adult son are less available to daughters, particularly to those who remain dependent on family funding for their studies. Shu’s account, in which her family would more readily accept a gay son than a lesbian daughter while contemplating forced marriage for her, is the participant statement of this pattern.

What the analysis claims, therefore, is not that lesbian women experience a greater quantity of Confucian pressure but that the pressure takes a form that is more temporally compressed, more relationally surveilled, and less escapable, and that these three properties are what carry the psychological consequences documented in Theme 3. This is a claim generated from eight accounts by women, and it awaits testing against samples that include gay men within a single design.

Two extensions to the minority stress framework follow from the results and are stated here rather than in Section 4, where the analytic task was to stay with participants’ accounts. The first concerns digital space. Peng’s loss of her WeChat community, and her account of the effort of ongoing self-censorship, describe a state-level enactment of stigma that operates through the interpersonal pathway by removing community and through the social-cognitive pathway by imposing a continuous monitoring load. The framework was formulated before pervasive platform surveillance and does not specify this mechanism, although it accommodates it without strain once specified. The second concerns time. Ying’s withdrawal from research applications in her second year describes anticipatory career foreclosure, in which the expectation of future discrimination produces present withdrawal from opportunity. The framework models the effect of anticipated rejection on current social behaviour but not on the imagined professional future, and educational settings, where present decisions determine later trajectories, are precisely where that omission matters.

The regional dimension identified in this study, the co-occurrence of material resource deprivation and intensified stigma in economically peripheral institutions represents a structural amplifier of risk of the psychological distress. Zhou et al. (40) document that social exclusion among LGB young adults in China produces self-reinforcing cycles of distress, where stigma generates withdrawal, isolation intensifies internalized stigma, and internalized stigma worsens mood, a dynamic particularly severe in residential educational environments. Addressing regional inequality requires policy reform that simultaneously targets geographic inequalities in educational funding (27, 28) and anti-LGBTQ stigma, as these two systems of disadvantage co-occur in the same populations and compound each other’s psychological effects.

### 5.2 Intersectional invisibility and its psychological implications

The mechanism of intersectional invisibility (9) has a specific psychological implication documented in this study; Ouyue’s single-axis framing of her disadvantage as a gender issue represents an adaptive defense that reduces the psychological cost of LGBTQ identity exposure while simultaneously preventing access to the LGBTQ community resources that are the primary evidence-based moderator of minority stress outcomes. Psychological intervention for lesbian and bisexual women in Chinese HEIs must therefore address both dimensions simultaneously: gender-based discrimination and sexuality-based stigma cannot be treated as parallel concerns, because their interaction produces self-reported psychological outcomes that neither single-axis intervention can adequately address. This finding directly challenges the design of existing equity initiatives in Chinese HEIs, which address gender and LGBTQ concerns in separate policy frameworks.

This also has implications for how researchers and clinicians ask about minority stress. If lesbian and bisexual women in high-stigma contexts tend to attribute compound disadvantage to the less-stigmatized identity category, studies using single-axis recruitment and measurement frameworks will systematically underestimate both the prevalence and severity of minority stress in this population. The finding that intersectional invisibility operates not only at the institutional level but at the level of self-perception suggests that future research should actively probe for compound identity stressors rather than relying on self-report of sexuality-based disadvantage alone.

### 5.3 From structural diagnosis to evidence-based psychological intervention

The minority stress model’s specification of protective factors directly identifies targets for institutional psychological intervention. Community connectedness is generated by formal recognition of LGBTQ student organizations: official endorsement provides an institutional signal reducing the expectation of rejection that drives chronic anxiety, consistent with Li et al.’s (5) finding that social support significantly moderates the minority stress pathway in Chinese LGB college students. Positive LGBTQ identity development requires LGBTQ-inclusive curricular content providing affirming frameworks for understanding one’s own identity countering the exclusively heterosexual sex education that currently leaves lesbian and bisexual women without conceptual resources to affirm their desires. Perceived institutional support is generated by enumerated non-discrimination policies: when institutions explicitly name sexual orientation and gender identity as protected characteristics, the expectation of rejection is reduced (44, 45).

These are the mechanisms; their implementation in Chinese HEIs requires a further step, because the institutional forms through which they are delivered elsewhere are largely unavailable here. Four pathways are feasible within existing structures. The first is the counselling centre. Every Chinese HEI operates a psychological health education and counselling centre under Ministry of Education requirements, staffed by counsellors who already undergo periodic in-service training. Sexual minority mental health can be added to that existing cycle as a clinical competence question rather than as a political one, and the immediate deliverable is modest: counsellors who do not treat same-sex attraction as the presenting problem, who do not disclose to advisers or families without consent, and who recognise the limits of their own training. The second is the student affairs system. The class advisers and counsellors who administer dormitory allocation, class affairs, and family liaison are the staff whose routine decisions produce the dormitory anxiety and the disclosure risk that participants described. Their existing training already covers crisis identification and student wellbeing, and non-discriminatory practice can be introduced there as an element of duty of care rather than as advocacy.

The third pathway is anonymity. Where visible services are not viable, low-threshold and anonymous channels are: written or online consultation without name registration, general wellbeing groups whose framing does not require identity disclosure as a condition of attendance, and referral lists that include community organisations without requiring the institution to endorse them. The fourth is the general clause. An institution that cannot enumerate sexual orientation in a policy document can still apply its existing student conduct rules on harassment and personal insult to homophobic conduct, and can say publicly that it does so. Enumeration is more protective, and the evidence base concerns enumerated policies specifically; consistent enforcement of a general clause is a second-best option that current conditions permit, and its limits should be stated rather than obscured.

None of these four pathways requires a change in national policy, an autonomous student organisation, or public institutional advocacy, and none produces the institutional signal that enumerated protection produces. They are offered as the interventions available under present constraints rather than as equivalents of the measures from which the underlying evidence is drawn. The evidence for their effectiveness in this specific context does not yet exist, and generating it is a more immediately useful research task than importing further findings from settings with legal protection.

LGBTQ-affirmative cognitive-behavioral therapy (CBT) represents the appropriate clinical-level complement to these structural interventions. Li et al. (5) demonstrated its effectiveness in reducing internalized homophobia and affective symptoms among Chinese LGB students. However, addressing internalized homophobia requires simultaneous reduction in the ongoing distal stressors that continuously replenish proximal stress processes: therapy that reduces internalized shame while the educational environment continues to produce shame-inducing interactions has limited and temporary psychological benefit. The implication for service design is clear: LGBTQ-affirmative counselling within HEIs must operate alongside, not as a substitute for, structural anti-stigma and anti-discrimination initiatives.

The political feasibility of these recommendations within China’s current governance context must be honestly appraised. Under Xi Jinping’s administration, LGBTQ civic space has been systematically restricted, university LGBTQ organizations closed, and LGBTQ content censored. Recommendations developed in Western contexts with legal LGBTQ protections cannot be uncritically transposed to this environment. Shu’s emphasis on “soft advocacy” incremental institutional changes that operate within rather than against existing political constraints, reflects the contextually embedded understanding that participants themselves brought to this question. When educators are perceived as supportive, LGBTQ students report significantly reduced victimization, improved mental health, and higher academic engagement (41, 42). Future research on the institutional conditions that enable incremental change should be designed with the risks of that documentation in view. Mapping the political opportunity structure of a specific university means describing, in a publicly indexed venue, which administrators have proved sympathetic, which channels have been used successfully, and which practices have survived scrutiny. In a governance environment that has closed student organisations and removed content, such a map is as usable by those who would close the openings as by those who would use them, and the individuals named or inferable within it carry the consequences. Research of this kind should therefore report mechanisms at a level of abstraction that does not identify institutions or individuals, should treat local intermediaries as requiring the same protection as participants, and should weigh whether findings whose principal value is tactical are better returned directly to the practitioners who can act on them than published in a form that also returns them to those who can foreclose them. The general point holds beyond this example: in high-stigma and low-protection settings, the visibility that ordinarily makes research useful can also make it dangerous, and that tension is a design consideration rather than a caveat.

## 6 Conclusion

This study provides a psychologically grounded account of the mechanisms through which lesbian and bisexual women in Chinese higher education experience, internalize, and are harmed by structural stigma and institutional discrimination. Guided by minority stress theory, Hatzenbuehler’s psychological mediation framework, and intersectionality theory and conducted through a reflexive thematic analysis approach that positioned researcher positionality as analytically generative rather than simply ethically declarable, the analysis demonstrates that the elevated risk of psychological distress of in this population is structurally produced, psychologically mediated, and institutionally addressable.

The five themes document the full psychological architecture of minority stress in this context: chronic anticipatory anxiety driven by hypervigilance; academic self-efficacy impairment driven by structural resource exclusion; internalized shame, affect dysregulation, and social isolation driven by enacted stigma and microaggression; compound psychological distress driven by intersectional invisibility; and attenuated outcomes where community resilience and institutional support are present. This architecture directly specifies the targets for intervention: enumerated non-discrimination policy; LGBTQ-affirmative psychological services; mandatory educator professional development; and formal recognition of LGBTQ student organizations.

The study also identifies two theoretical contributions to the minority stress literature. First, the culture-specific stressor of marriage pressure is gendered in its distribution falling more intensely on lesbian and bisexual women than on gay men in the Confucian context, in a way that the existing framework, developed with Western male samples, does not specify. Second, intersectional invisibility operates not only at the institutional policy level but at the level of lesbian and bisexual women’s own self-perception in high-stigma contexts, with direct implications for how minority stress research measures and recruits participants. The psychological distress of lesbian and bisexual women in Chinese higher education is not inevitable. It is the product of specific, identifiable structural conditions that universities have both the capacity and, the data suggest, the responsibility to address.

### 6.1 Critical reflection

This study makes a meaningful contribution to the psychological literature on LGBTQ mental health in non-Western, high-stigma contexts. However, several critical reflections are warranted.

First, while minority stress theory provides well-validated conceptual scaffolding, its Western individualist origins raise questions about its direct applicability to the Chinese educational context, where the self is constituted relationally (53) rather than individually. Stigma experienced through family shame and filial obligation may produce psychological harm through mechanisms that Meyer’s original formulation does not fully specify. This study applies the framework with this limitation explicitly acknowledged, but future theoretical work should develop culturally adapted extensions incorporating collectivist self-construal and relational obligation as distinct structural variables within the minority stress model.

Second, the study’s analytical framework while comprehensively covering psychological mechanisms of harm gives less analytical weight to resilience than to deficit. The primary frameworks (minority stress, stigma mediation) direct attention toward distress rather than toward the adaptive strategies, community strengths, and identity affirmation processes that lesbian and bisexual women in China actively deploy. This is an inherent tension in psychiatric research with marginalized populations: the clinical utility of documenting harm risks reproducing the pathologizing gaze that the study simultaneously critiques. A future study combining minority stress theory with a strengths-based framework, for example, positive identity development theory (55) would provide a more complete account of psychological functioning in this population.

Third, while this study adopted reflexive thematic analysis in its epistemological commitments, the reflexivity enacted in the analysis was necessarily partial, the researcher’s positionality as an outsider to LGBTQ experience and an insider to the Chinese cultural context created interpretive asymmetries that cannot be fully resolved through procedural safeguards. The member-checking conducted with three participants provided a partial corrective, but a fully collaborative or participatory research design, in which LGBTQ-identifying researchers or community members were co-analysts would have strengthened the interpretive authority of the findings substantially. More fundamentally, future research involving LGBTQ participants in high-stigma contexts should build consultation with LGBTQ researchers or specialist organisations into the study design phase rather than treating it as an optional enhancement. Such consultation is not merely an ethical improvement but a methodological safeguard: it provides an external check on design decisions — including sampling and inclusion criteria, that researcher reflection alone may not adequately scrutinise.

### 6.2 Research limitations

Eight participants across four province-level regions provide sufficient depth for qualitative thematic analysis but limits breadth. The sample cannot represent the full diversity of lesbian and bisexual women’s experiences across China’s enormous geographic, institutional, and socioeconomic range. Participants were predominantly enrolled in arts, humanities, and social science disciplines; the experiences of lesbian and bisexual women in STEM fields where gender-based stereotypes about academic aptitude are most pronounced and where masculine institutional cultures may compound sexuality-based stigma are not captured.

Recruitment bias is a significant concern. Purposive sampling required participants to self-identify as LGBTQ and agree to be interviewed about their experiences of discrimination. This systematically excludes lesbian and bisexual women who have not disclosed their identity, who do not identify with LGBTQ labels, or who experience their situation as too psychologically unsafe to discuss with a researcher. Participants who agreed to be interviewed may be more psychologically resilient, more socially connected within LGBTQ networks, or more politically aware than the broader population, meaning this study may underestimate psychological distress severity among the most marginalized and isolated members of this population.

The cross-sectional design prevents tracing the developmental trajectory of minority stress processes. It is not possible from this data to determine when internalized homophobia developed, how it has changed over time, or how it interacts with transitions across different institutional environments. Longitudinal designs tracking lesbian and bisexual women’s psychological experiences from secondary school entry through higher education and into employment would substantially deepen understanding of how minority stress accumulates and whether HEI experiences produce lasting psychological consequences.

The study’s sample comprises cisgender lesbian and bisexual women only. The experiences of transgender women in Chinese HEIs, who face additional layers of institutional pathologization, including China’s continued classification of gender incongruence as a mental disorder under CCMD-3 and the legal prohibition on gender marker changes — are therefore not captured in this study. Their psychological experiences are likely qualitatively distinct from and compounded beyond those of cisgender participants. Future research should specifically recruit transgender women in Chinese HEIs to enable analysis of their distinct minority stress processes, and should ensure that trans-affirmative methodological consultation is built into the study design from the outset.

Finally, the study makes reference to clinical constructs internalized homophobia, shame-based affect dysregulation, depressive symptoms inferred from qualitative interview accounts rather than assessed through validated psychometric instruments. The psychological framing of the findings should be read as theoretical specification rather than clinical diagnosis. Future research combining reflexive thematic analysis with validated measures such as the PHQ-9 (51), GAD-7 (56), and the Internalized Homophobia Scale (54) would provide both the rich contextual understanding that qualitative methods afford and the clinical precision that psychological research requires.

## Data Availability

The original contributions presented in the study are included in the article/Supplementary material, further inquiries can be directed to the corresponding author.

## Supplementary material

SUPPLEMENTARY FILE 1 English-language interview protocol used in data collection, encompassing five theoretically grounded domains.

SUPPLEMENTARY FILE 2 Chinese originals and English translations of all data extracts reported in Section 4, provided for reader verification of translation accuracy.

SUPPLEMENTARY FILE 3 Complete hierarchical coding tree showing all 87 initial codes, candidate themes, and final five themes.
