# Supplementary material for: Psychological distress, stigma, and intersectional marginalization in lesbian and bisexual cisgender women in Chinese higher education: a thematic analysis guided by minority stress theory
Source: Front Public Health. 2026 Aug 28;14:1893495. doi: 10.3389/fpubh.2026.1893495 (PMC13561772; doi:10.3389/fpubh.2026.1893495)
Supplement: Supplementary file 1 [file Supplementary_file_2.pdf]

Supplementary File 2: Chinese-English Quotations & Cultural Notes

Note: All quotations are verbatim from interview transcripts. Cultural glosses are provided for terms whose semantic weight is not carried by their nearest English equivalent.

Theme 1 hilighted in pink, theme 2highlighted in green, theme 3 hilighted in blue, theme 4 hilighted in orange, theme 5 hilighted in grey.

Chinese Original	English Translation	Cultural Note
“我感觉，性别不平等是指一些人仅仅因为性别而受到的不平等待遇、期望和机会。这是一种特别普遍情况吧，男性、女性或者 LGBTQ 而受到不同或者差异性的对待，就是弱势群体。” Theme 1	“Gender inequality is all unequal treatments, expectations, and opportunities that some people go through just because of their gender. It is a situation whereby these people are treated differently on the basis they are male, female, or even LGBTQ, making them a disadvantaged group.”	—
“我过去常常通过微信上的 LGBTQ 群体寻找社区。我们的表达都可能比较直白，当那些账号被关闭时，我失去了一个可以做我自己的空间。现在我更加小心我在网上写什么或分享什么。那种小心很累人。”	“I used to find community through LGBTQ groups on WeChat. When those accounts were closed, I lost a space where I could be myself. Now I am more careful about what I write or share online. That carefulness is exhausting.”	数字监控 (shùzì jiānkòng) = digital surveillance
“我身边的的 LGBTQ 女性更加沉默，其实他们挺害怕表达自己。”	“LGBTQ women in my environment were more silent and afraid to express themselves.”	自我沉默 (zìwǒ chénmò) = self-silencing as a protective strategy

“一个男同学告诉我‘一旦我试过男人，我就不会再是女同性恋了’。这很羞辱人。另外，人们把 HIV 和其他坏事归咎于同性恋群体，认为我们破坏了社会和谐。其实，LGBTQ 是一个在社区中面临高度歧视的群体，因为他们被认为是不道德的，我们学习中这样的歧视也存在很多。”<br>Theme 3	“One male student told me 'once I tried a man, I would not be lesbian anymore'. It was humiliating. Also, people blamed HIV and other bad things on us, because they agreed that we had disrupted social harmony. LGBTQ is a group in the community that faces high discrimination since they are considered to be immoral, and this can affect even their academic performances.”	-
“我父母一直给我发关于扭转治疗的文章，他们觉得这是一个心理问题。我感觉我回不了家了。有时候我不想和父母有任何对话。我现在害怕和他们说话——他们总是想教育我。”	“My parents keep sending me articles about conversion therapy, as they think this is a psychological problem. I feel like I cannot go home. Sometimes I would not want to have any conversation with my parents. I am afraid to talk with them now — they always want to educate me.”	扭转治疗 (niǔzhuǎn zhìliáo) = conversion therapy
“我的老师问我，我是不是在假装是女同性恋，或者这只是青春期的叛逆行为。我知道她不是想伤害我，但是……”	“My supervisor asked me whether I was pretending to be a lesbian or whether it was just adolescent rebellious behaviour. I knew she was not trying to hurt me, but…”	微侵犯 (wēi qīnfàn) = microaggression
“没有任何一件事严重到可以举报。但加在一起，它们让我觉得我不属于这里。我开始怀疑我是不是把事情理解错了，是不是我太敏感了。那种自我怀疑比任何一句评论都糟糕。”	“No one incident was terrible enough to report. But together they made me feel that I did not belong. I started to wonder whether I was reading the situation wrong, whether I was too sensitive. That self-doubt was worse than any single comment.”	“煤气灯效应” (méiqìdēng xiàoyìng) = gaslighting effect of accumulated microaggressions

“直女有时候会担心或害怕我们，如果我们住在同一个宿舍的话，而男同学认为我们的行为不正常。在课堂上，他们用这种看法来孤立我们。在小组作业中，我通常去那个人不够的小组，我选择最后加入那个组，这样似乎更容易被接受。”	“Straight women are sometimes, worried or afraid of us if we live in the same dormitory, and male students think our behaviour is not normal. In the classroom, they use this perception to isolate us. In group assignments, I usually go to the group that is not full, I chose to join lately the group, it seems more acceptable.”	宿舍排斥 (sùshè páichì) = dormitory-based social exclusion
“我不知道该怎么告诉我的亲戚和父母，他们可能会真的很生气或困惑，可能会逼我像别人一样，到了合适的年龄（毕业后）就嫁给一个男人。但对于我的弟弟，他们接受他是同性恋。”	“I do not know how to tell my relatives and parents, they could be really angered or confused, and may force me to act like others, like marry a man at the right age (after graduation). But for my little brother, they accepted that he is gay.”	逼婚 (bī hūn) = coerced marriage<br>孝 (xiào) = filial obligation
“男学生被给予更多的机会，比如领导角色，甚至在学术旅行资助和研究资助中获得更大的份额。这些行为通常会阻止女性和 LGBTQ 学生参与这类活动。”<br>Theme 2	“Male students are given more opportunities such as leadership roles, and they are even given larger shares during academic trips' funding and research funding. These actions usually discourage female and LGBTQ students from participating in such activities.”	资源剥夺 (zīyuán bōduó) = resource deprivation
“城市里的教育资源在性别上没有什么差异，但贫困地区的教育资源偏向男性，而且对 LGBTQ 女性的容忍度有限，那些女性被描述为‘有病’。”	“There is no difference in the skewing of educational resources for gender in cities, but educational resources in poorer areas are skewed in favour of males, and there is limited tolerance of LGBTQ women — those women are described as sick.”	“有病” (yǒu bìng) = pathologizing language

“我大二的时候就停止申请研究职位了。我觉得一旦我承认了自己的性取向，我的机会就小了。我的身份使我在控制那些机会的教师面前变得隐形。”	“I stopped applying for research positions in my second year. I did not think I had a realistic chance, once I admitted my sexuality. My identity made me invisible to the faculty who controlled those opportunities.”	预期性职业终止 (yùqī xìng zhíyè zhōngzhǐ) = anticipatory career foreclosure
“作为一个女性会影响别人如何看待我。这不仅仅是作为一个女人的问题；而是关于我的性取向如何与之交叉，很独特。”<br>Theme 4	“Being a queer woman affects how others perceive me. It's not just about being a woman; it's about how my sexual orientation intersects with that, leading to unique experiences.”	交叉性 (jiāochā xìng) = intersectionality
“我可以看到事情在慢慢变化。现在有更多的学生意识到 LGBTQ 问题，比我刚开始的时候多。但是制度还没有跟上。意识存在，但没有得到保护。”	“I can see that things are slowly changing. There are more students who are aware of LGBTQ issues now than there were when I started. But the institutions have not caught up. The awareness is there but it is not protected.”	个体意识 vs 制度保护 (gètǐ yìshí vs zhìdù bǎohù)
“当人们讨论性别问题时，他们只把我看作一个女人，而当他们讨论 LGBTQ 权利时，他们只把我看作一个同性恋。从来不会同时作为两者。”	“People only see me as a woman when they discuss gender issues, and only as gay when they discuss LGBTQ rights. Never as both.”	交叉性隐形 (jiāochā xìng yǐnxíng) = intersectional invisibility
“学校可以默许性少数俱乐部的存在，或者通过软性倡导将其推广为官方认可。发声是被不公平对待的人有效地表达他们经历的方式，以便让更多人	“Schools can acquiesce to the existence of sexual minority clubs or promote them as official endorsements with soft advocacy. Being vocal is an effective way for people who have been treated unfairly to speak out about their experiences so that more people realise there is a problem.”	软性倡导 (ruǎn xìng chàngdǎo) = soft advocacy

意识到问题的存在。”<br>Theme 5		
“有时我觉得问题不在于老师不知道我们的存在。他们知道。问题在于知道我们的存在和正式承认我们是两件非常不同的事情。在中国，那个知道和说出来之间的差距，就是我们生活的地方。如果老师包容度高，哪怕只是在课堂上稍微缩小一点这个差距，就意味着我们不必继续假装我们彼此之间没有归属感。一个关于性别歧视培训项目可能不会改变法律。但它可能会让被看见这件事变得稍微不那么危险。”	“Sometimes I think the problem is not that teachers do not know we exist. They do. The problem is that knowing we exist and acknowledging us officially are two very different things. In China, that gap, between knowing and saying, is where we live. If teachers were trained to at least close that gap in the classroom, even a little, it would mean we did not have to keep pretending that we do not belong to each other. Right now, we find each other quietly, carefully. A training programme would not change the law. But it might make it slightly less dangerous to be seen.”	知道与承认的差距 (zhīdào yǔ chéngrèn de chājù) = gap between knowing and acknowledging
“我在网上找到了一个社交群，我可以毫无恐惧地谈论我的身份。那是一个小团体。我们对使用什么平台很小心。它帮助我感觉不那么孤独。”	“I found an online community where I could talk about my identity without fear. It was a small group. We were careful about what platform we used. It helped me feel less alone.”	数字避难所 (shùzì bìnánsuǒ) = digital refuge

Note: Theme tags correspond to the five themes in the manuscript: Theme 1 (Awareness of Structural Inequality), Theme 2 (Institutional Resource Deprivation), Theme 3 (Enacted and Internalized Stigma), Theme 4 (Intersectional Invisibility), Theme 5 (Attenuated Outcomes with Support). All translations were verified by a second bilingual reviewer.
