# Supplementary material for: Psychological distress, stigma, and intersectional marginalization in lesbian and bisexual cisgender women in Chinese higher education: a thematic analysis guided by minority stress theory
Source: Front Public Health. 2026 Aug 28;14:1893495. doi: 10.3389/fpubh.2026.1893495 (PMC13561772; doi:10.3389/fpubh.2026.1893495)
Supplement: Supplementary file 2 [file Supplementary_file_3.pdf]

Coding Tree: Reflexive Thematic Analysis Process

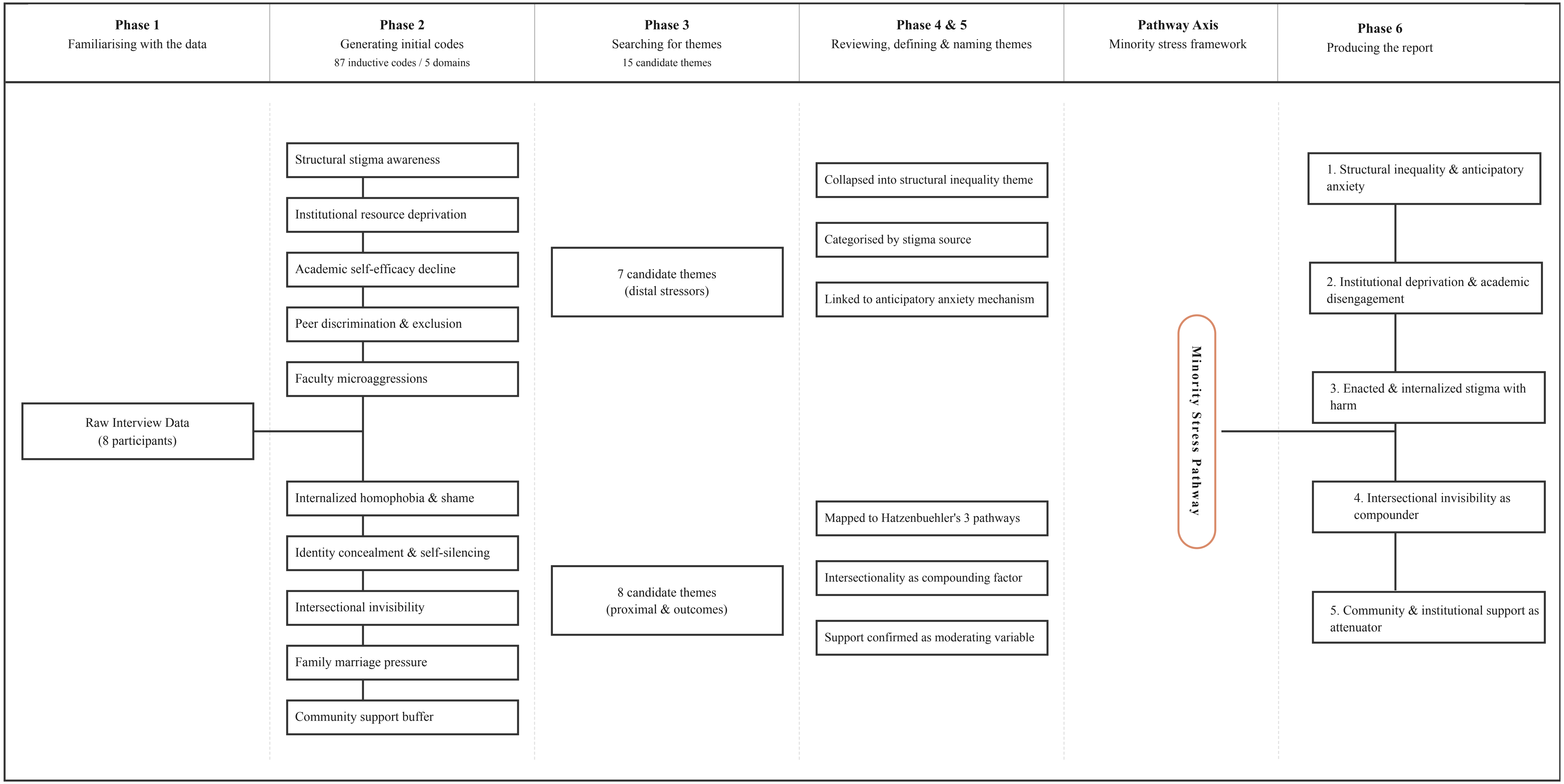

Note. The coding process follows Braun & Clarke's (2022) six-phase reflexive thematic analysis framework.
