# Supplementary material for: Psychological distress, stigma, and intersectional marginalization in lesbian and bisexual cisgender women in Chinese higher education: a thematic analysis guided by minority stress theory
Source: Front Public Health. 2026 Aug 28;14:1893495. doi: 10.3389/fpubh.2026.1893495 (PMC13561772; doi:10.3389/fpubh.2026.1893495)
Supplement: Supplementary file 3 [file Supplementary_file_1.DOCX]

Psychological Distress, Stigma, and Intersectional Marginalization Among LGBTQ Women in Chinese Higher Education

**Overview**

This semi-structured interview protocol was developed specifically for the study that aimly stated. It was designed to explore the psychological and psychiatric dimensions of LGBTQ women’s experiences in Chinese higher education institutions (HEIs), guided theoretically by Meyer’s (2003) minority stress model, Hatzenbuehler’s (2009) psychological mediation framework, and Crenshaw’s (1989) intersectionality theory.

Interviews were conducted in Mandarin (Chinese), which lasted approximately 60–90 minutes each. The protocol was piloted with two LGBTQ-identifying individuals not included in the main sample, and revised interview questions and themes orfer for clarity and cultural appropriateness prior to use with study participants. Only audio recordings were made with informed consent. The English-language version presented here was produced by the primary researcher and reviewed by a bilingual (English-cerficated) colleague from the English and translation.

**Administrative Information**

*Language of administration*: Mandarin Chinese (this document is an English-language translation).

*Estimated duration*: 60–90 minutes.

*Format:* The study format consisted of audio-recorded semi-structured interviews with the individuals. The purpose is to avoid raising any feelings of insecurity in the participants and to protect their identity. To protect the privacy of participants and avoid raising their stress, it is not allowed to conduct video recording during the fieldwork.

*Setting*: Online interview ( (encrypted video platform) or face-to-face, based on participant preference, in this study 3 participants chose to be interviewed online, seven were interviewed in-person by face-to-face.

*Piloting*: Conducted with two LGBTQ-identifying individuals not included in the main sample. Questions were revised for clarity and cultural sensitivity following piloting.

**Opening Script**

The following statement was read to each participant at the start of the interview:

*‘Thank you for agreeing to take part in this study. Our conversation will focus on your experiences as an LGBTQ woman in Chinese higher education. We will discuss topics such as gender inequality, experiences of discrimination or stigma, and how these experiences may have affected you personally and academically. We will also discuss how you feel about your own identity, and any internal pressures or conflicts you may have experienced as a result of your LGBTQ identity and the social context you live in. There are no right or wrong answers. I am interested in your own perspective. You do not have to answer any question you are uncomfortable with. You may stop the interview at any time, without any consequence, simply by telling me you wish to stop. With your permission, I will audio-record this interview so that I can produce a written transcript. No video recording will be made. The recording will be stored securely and will not be shared with anyone outside the research team.’*

**Domain 1: Awareness and Cognitive Appraisal of Gender Inequality and Stigma**

*Theoretical basis: Distal stressor recognition; expectation of rejection (Meyer, 2003); social-cognitive pathway activation (Hatzenbuehler, 2009).*

*Purpose: To explore how participants understand and cognitively appraise their stigmatised position. Chronic awareness of structural inequality is itself a proximal stressor generating anticipatory anxiety.*

1. ***How would you describe gender inequality in Chinese higher education?***

- *What forms does it take in your day-to-day experience?*
- *Do you think LGBTQ students experience this inequality differently from heterosexual students?*

2. ***When did you first become aware of your position as an LGBTQ woman at university?***

- *What made you aware of it?*
- *How did that awareness change how you behaved or felt?*

3. ***How does being aware of your LGBTQ identity affect how you move through daily university life?***

- *Are there spaces or situations where you feel more alert or guarded?*
- *Does this alertness feel tiring or burdensome?*

4. ***Have you noticed differences in how LGBTQ women are treated compared to gay men at your institution?***

- *Can you give a specific example?*
- *How do you make sense of that difference?*
- *Do you feel that spaces or movements advocating for gender equality, or for LGBTQ rights, fully represent someone in your position — someone who is both a woman and LGBTQ? Or do you find yourself falling between them?*
- *Have you ever felt invisible — not fully seen or recognised — in a context that was supposed to be a supportive space for you?*

5. ***How has awareness of stigma affected how you present yourself in academic settings?***

- *Do you adjust your behaviour or self-presentation around certain people or in certain contexts?*

**Domain 2: Personal and Academic Experiences of Discrimination and Their Affective and Behavioural Sequelae**

*Theoretical basis: Enacted stigma as distal stressor; minority stress pathway from external discrimination to internal harm (Meyer, 2003); social-cognitive and interpersonal pathways (Hatzenbuehler, 2009).*

*Purpose: To document specific discrimination experiences and their psychological and academic sequelae, including effects on concentration, motivation, and peer relationships.*

1. ***Have you experienced discrimination or unfair treatment at university because of your gender identity or sexual orientation?***

- *Can you describe what happened?*
- *Who was involved peers, teaching staff, or administrative staff?*
- *How did you feel during and after that experience?*

2. ***Have you witnessed discrimination against other LGBTQ students?***

- *What did you observe?*
- *How did witnessing it affect you personally?*

3. ***How have your experiences as an LGBTQ woman affected your academic engagement or performance?***

- *Have there been times when stigma-related stress made it harder to concentrate or attend class?*
- *Have your relationships with peers or instructors been affected?*

4. ***Are there specific aspects of university life where your LGBTQ identity creates particular difficulties?***

- *For example: dormitory arrangements, group work, classroom participation, or social events?*
- *Can you describe a specific situation?*

5. ***Have you ever experienced microaggressions, like comments or actions that were not openly hostile but still felt invalidating or dismissive of your identity?***

- *Who made these comments or took these actions?*
- *How did they affect you emotionally at the time, and afterwards?*

6. ***Have you had to manage or limit your use of social media or online spaces because of your LGBTQ identity?***

- *Has state censorship or platform restrictions affected your access to LGBTQ community spaces online?*
- *How has this affected your sense of connection or support?*
- *If an online LGBTQ community or group you relied on was closed or restricted, what did that feel like? What did you lose when that space disappeared?*

**Domain 3: Internalized Stigma Processes, Including Shame, Self-Concealment, and Identity Conflict**

*Theoretical basis:Proximal stressors in the internalized homophobia, identity concealment, anticipatory rejection (Meyer, 2003); emotional regulatory pathway; shame and affect dysregulation (Hatzenbuehler, 2009).*

*Purpose: To explore how external stigma has been absorbed as internal psychological harm, including shame, self-silencing, and conflict between LGBTQ identity and cultural expectations.*

1. ***How do you feel about your LGBTQ identity when you are on your own, separate from how others react to it?***

- *Has this feeling shifted over time?*
- *Are there moments when you feel negatively toward yourself because of your identity? What tends to trigger that?*

2. ***Have you ever felt the need to hide your LGBTQ identity at university?***

- *What made concealment feel necessary?*
- *What does managing what you disclose feel like on a day-to-day basis?*
- *Has concealment affected your relationships or your sense of authenticity?*

3. ***Have you experienced tension between your LGBTQ identity and your family role or cultural expectations?***

- *Can you describe what that tension feels like?*
- *How does it affect your emotional wellbeing?*

4. ***Have you experienced pressure to conform to heterosexual norms, including expectations around marriage?***

- *Where does this pressure come from family, peers, or broader social expectation?*
- *How do you experience this pressure emotionally?*
- *Do you feel this pressure is different for you as a woman compared to gay men you know?*

5. ***Has your LGBTQ identity ever led you to question your academic plans or career aspirations?***

- *Have you considered avoiding certain career paths or workplaces because of anticipated discrimination?*
- *How does this uncertainty affect your motivation or sense of future?*

***Domain 4: Perceived Institutional Climate and Its Moderating Effects on Psychological Wellbeing***

*Theoretical basis: Structural conditions as distal stressors or protective factors; perceived institutional support as moderator of minority stress (Meyer, 2003; Hatzenbuehler, 2009); regional inequality and resource deprivation.*

*Purpose: To explore how institutional environments, it including policies, staff attitudes, and resource allocation, it moderate psychological outcomes for LGBTQ women.*

1. ***How would you describe your university’s climate toward LGBTQ students?***

- *Does the institution acknowledge the existence of LGBTQ students in any formal or informal way?*
- *Are there policies, statements, or resources that signal how the institution views LGBTQ identity?*

2. ***Have you ever sought support from your institution related to your experiences as an LGBTQ student?***

- *If yes: what was that experience like? Did the support feel affirming of your identity?*
- *If no: what has prevented you from seeking support?*

3. ***Do you feel your geographic location affects your experience as an LGBTQ student?***

- *For example, is being at an institution in a major city different from being in a smaller regional city?*
- *In what specific ways does location shape the stigma or support you encounter?*

4. ***Have you noticed inequalities in how educational resources, leadership roles, or research opportunities are distributed?***

- *Do you feel your LGBTQ identity, or your gender, affects your access to these opportunities?*
- *How has this affected your sense of belonging or academic confidence?*

5. ***Are you aware of any LGBTQ student organizations or community groups at your institution or in your city?***

- *Have these groups been accessible to you?*
- *Has the situation changed in recent years — for example, have groups been closed or restricted?*

**Domain 5: Coping Strategies, Help-Seeking Behaviour, and Resilience Resources**

*Theoretical basis: Protective factors in minority stress model — community connectedness, positive identity, social support (Meyer, 2003); interpersonal pathway moderation; institutional support as structural intervention.*

*Purpose: To explore what moderates or buffers psychiatric risk, including community belonging, individual coping, and institutional conditions that support resilience.*

1. ***When you experience stress related to your LGBTQ identity, what do you do to cope?***

- *Are there people, spaces, or communities that provide meaningful support?*
- *Are there online or offline LGBTQ networks that are important to you?*
- *Have you had to find alternative spaces following restrictions on LGBTQ groups or content?*

2. ***Have you ever sought mental health support, such as counselling, therapy, or peer support-related to your LGBTQ identity?***

- *If yes: was the support affirming of your identity? Was it helpful?*
- *If no: what has shaped your decision not to seek support?*

3. ***What aspects of your identity, relationships, or community give you a sense of strength?***

- *Has your LGBTQ identity ever been a source of positive meaning, connection, or pride?*
- *Have you developed ways of affirming your identity despite the social context?*

4. ***What changes at your institution would most meaningfully improve the experience of LGBTQ women students?***

- *In terms of policy, services, staff training, or culture?*
- *Are there things you feel are realistically achievable within the current political context in China?*

5. ***Is there anything about your experience that you feel has not been captured in our conversation?***

- *Is there anything you would like to add or correct?*

**Closing Script**

*‘Thank you very much for your time and for sharing your experiences with me. Your participation is greatly valued. All information you have shared will be kept strictly confidential. You will not be identifiable in any published output. If any part of our conversation has brought up difficult feelings, you are not obligated to continue thinking about these topics. I am going to share with you a list of mental health resources available in your province. You are welcome to contact me with any questions about the study.’*

**Note on Protocol Administration**

This protocol is semi-structured. The five domains and core questions provided a consistent framework across interviews. However, the researcher had flexibility to adjust the order of questions, rephrase them, and introduce additional probes in response to what participants raised.

The follow-up probes listed are illustrative rather than exhaustive. The researcher did not press participants on aspects of their identity they had not volunteered. Participants were reminded of their right to decline any question or withdraw at any point without consequence.

Where participants disclosed acute distress, the researcher paused the interview and checked in on their wellbeing before proceeding. Mental health resources were provided to all participants at the close of the interview, regardless of whether distress was disclosed.
